# Active DNA demethylation upstream of rod-photoreceptor fate determination is required for retinal development

**DOI:** 10.1371/journal.pbio.3003332

**Published:** 2025-08-04

**Authors:** Ismael Hernández-Núñez, Alaina Urman, Xiaodong Zhang, William Jacobs, Christy Hoffmann, Ellen G. Harding, Shiming Chen, Meelad M. Dawlaty, Philip A. Ruzycki, John R. Edwards, Brian S. Clark

**Affiliations:** 1 John F. Hardesty, MD Department of Ophthalmology and Visual Sciences, Washington University School of Medicine, St. Louis, Missouri, United States of America; 2 Division of Oncology, Department of Medicine, Washington University School of Medicine, St. Louis, Missouri, United States of America; 3 Department of Developmental Biology, Washington University School of Medicine, St. Louis, Missouri, United States of America; 4 Ruth L. and David S. Gottesman Institute for Stem Cell and Regenerative Medicine Research, Albert Einstein College of Medicine, Bronx, New York, United States of America; 5 Department of Genetics, Albert Einstein College of Medicine, Bronx, New York, United States of America; 6 Department of Developmental and Molecular Biology, Albert Einstein College of Medicine, Bronx, New York, United States of America,; 7 Department of Genetics, Washington University School of Medicine, St. Louis, Missouri, United States of America; New York University, UNITED STATES OF AMERICA

## Abstract

Retinal cell fate specification from multipotent retinal progenitors is governed by dynamic changes in chromatin structure and gene expression. Methylation at cytosines in DNA (5mC) is actively regulated for proper control of gene expression and chromatin architecture. Numerous genes display active DNA demethylation across retinal development; a process that requires oxidation of 5mC to 5-hydroxymethylcytosine (5hmC) and is controlled by the ten-eleven translocation (TET) methylcytosine dioxygenase enzymes. Using an allelic series of conditional TET enzyme mutants in mice, we determine that DNA demethylation is required upstream of NRL and NR2E3 expression for the establishment of rod-photoreceptor fate. Using histological, behavioral, transcriptomic, and base-pair resolution DNA methylation analyses, we establish that inhibition of active DNA demethylation results in global changes in gene expression and methylation patterns that prevent photoreceptor precursors from adopting a rod-photoreceptor fate, instead producing a retina in which all photoreceptors specify as cones. Our results establish the TET enzymes and DNA demethylation as critical regulators of retinal development and cell fate specification, elucidating a novel mechanism required for the specification of rod-photoreceptors.

## Abstract 2

TET enzymes remove DNA methylation markers, but the role of this process in retinal development is not clear. This study shows that these enzymes are required for photoreceptor cells to initiate the genetic program to become rods instead of cones, and for maturation of the mouse retina.

## Introduction

The generation of the diverse array of retinal cell types is orchestrated through the active regulation of cell fate specification from a single pool of multipotent retinal progenitor cells (RPCs) [1–3]. Retinal cell fate specification across development is modulated by dynamic changes in chromatin structure and gene expression patterns to facilitate the temporal specification of retinal cell fates. The past few decades of work have identified transcription factors and gene regulatory networks (GRNs) that bias the temporal specification of retinal cell fates [4–18]. However, the exact mechanisms by which these transcription factors are expressed and their role in controlling specification of major cell type classes and specification of the >120 mouse retinal cell subtypes remains unclear [19–23].

Temporal and cell type-specific epigenetic modifications, including histone modifications, chromatin accessibility, and DNA methylation patterns bias chromatin remodeling and gene transcription for the specification of retinal cell fates [24–27]. In particular, DNA methylation profiles are temporally dynamic and display cell-type-specific DNA methylation patterns [27–31].

**Fig 1:**
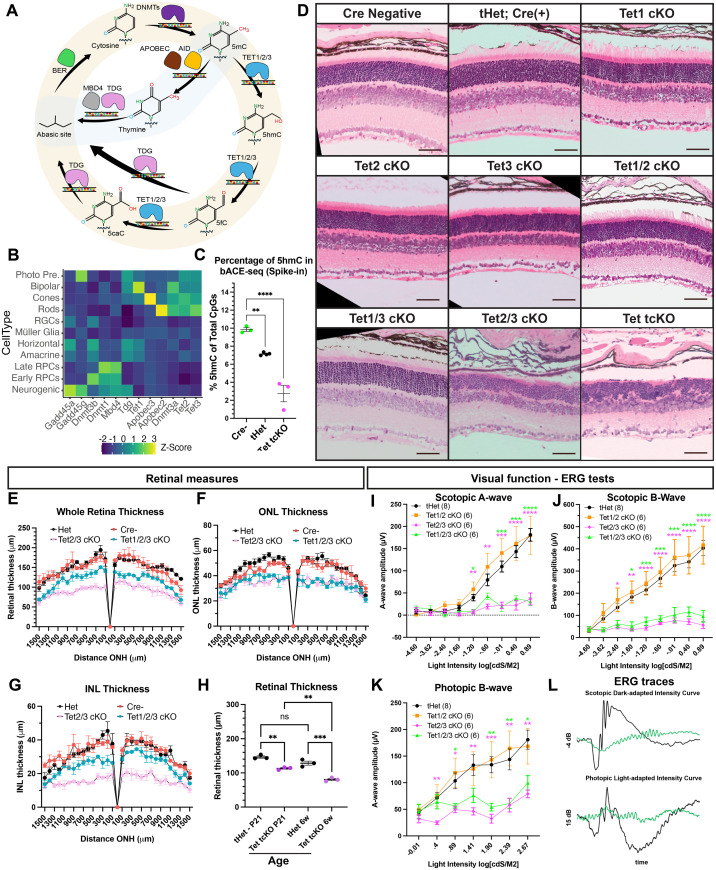
The TET enzymes are required for retinal development and visual function. **(A)** Active DNA methylation cycle—(**i)** 5mC is added by DNMTs. (**ii)** The TET enzymes oxidize 5mC to 5hmC. **(iii)** 5hmC is converted to 5fC and 5caC by the TET enzymes, followed by conversion back to cytosine by TDG and the base-excision repair pathway. (**iv)** Alternatively, APOBEC converts 5mC to thymine, causing DNA mismatch. **(B)** Expression of DNA demethylation pathway components is enriched in photoreceptors. single-cell RNA-sequencing (scRNAseq) data from [14]. **(C)** bACE-seq quantification of 5hmC across P21 retinas in Cre−, tHet, and Tet tcKO animal models. Statistics represent results of a One-way ANOVA followed by a Dunnett’s Multiple Comparisons test (** *p* < 0.01; **** *p* < 0.0001). **(D)** H&E staining of an allelic series of TET conditional P21 mutants. **(E)** Whole retina, **(F)** outer nuclear layer (ONL) and **(G)** inner nuclear layer (INL) thickness measures at different eccentricities from the optic nerve head (ONH). Results display the mean + SEM for *n* = 3 for each genotype in P21 retina. **(H)** Graph showing the comparison in the mean retinal thickness of control and Tet tcKO retinas between P21 and 6 weeks old retinas. Results display the mean + SEM for *n* = 3 for each genotype. Statistics are the result of a Two-way ANOVA with, followed by a Tukey’s multiple comparisons test. ns: nonsignificant; ** *p* < 0.01, *** *p* < 0.001; **** *p* < 0.0001. **(I–K)** Visual function testing as measured by full-field electroretinogram (ERG) indicating scotopic A-wave, scotopic B-wave and photopic B-wave amplitudes across different light intensities. Statistics are the result of a Two-way ANOVA with Geisser–Greenhouse correction, followed by Dunnett’s multiple comparison test to tHet controls. * *p* < 0.05; ** *p* < 0.01; *** *p* < 0.001; **** *p* < 0.0001. **(L)** Examples of ERG scotopic dark-adapted intensity (−4 dB) and photopic light-adapted intensity (15 dB) traces comparing tHet and Tet1/2/3 cKO retinas. Abbreviations: Neuro, Neurogenic RPCs; Photo Pre, Photoreceptor Precursor Cells; BER, base-excision repair; DNMTs, de-novo methyltransferases; TDG, thymine DNA glycosylase; Tets, tet-eleven translocation methylcytosine dioxygenases. Scale bars: 100 µm. Data files for graphs available in S1 Data.

DNA methylation is established through the addition of a methyl group to the 5 position of cytosine residues [5-methylcytosine (5mC), 32–35]. Both enzymatic and passive processes regulate the methylation status of DNA. DNA methylation is promoted by de-novo methyltransferases (DNMTs), including DNMT1, DNMT3a, and DNMT3b. Hypermethylation of promoters or enhancer sequences is correlated with reduced gene expression [35–36] and has an important role during development, aging, and disease [33]. However, many regulatory elements, including enhancers and promoters, undergo a transition from hypermethylation early in development to hypomethylation in mature cell types [37–45]. The removal of 5mC—DNA demethylation—is regulated via three distinct mechanisms; (1) a passive, DNA replication-dependent manner whereby methylation is reduced by 50% after each round of DNA synthesis; (2) an active process mediated by the ten-eleven translocation (TET) methylcytosine dioxygenases, TET1, TET2, and TET3 [46–48] (Fig 1A); or (3) conversion of 5mC to 5hmC by the TET enzymes and subsequent passive loss of 5hmC during DNA replication and cell division. 5hmC is further oxidized by the Tet enzymes to produce 5-formilcytosine (5-fC) and 5-carboxylcytosine (5-caC) [48] which are then converted back to cytosine by Thymine DNA-Glycosylase (TDG) and the base-excision repair (BER) pathway (Fig 1A) [43,49–53]. In addition to being an intermediate during demethylation, 5hmC is a stable mark that controls gene transcription, RNA splicing, or local control of histone modifications and chromatin remodeling [54]. 5hmC has an important regulatory role during both nervous system development and aging [55,56]; however, to date, the nucleotide-specific localization and significance of 5hmC deposition in retinal cell fate specification remains undetermined.

Genome-wide profiling of DNA methylation during retinal development has determined the temporal dynamics [27] and cell type-specific signatures of methylated DNA sequences [28–31], with 3%–38% of genes displaying an inverse correlation of local DNA methylation and RNA transcript expression [27]. Numerous gene promoters and gene bodies of rod and cone-photoreceptor genes are methylated in RPCs but display low DNA methylation levels and increased chromatin accessibility in mature photoreceptors [27,29,30,57]. This has led to the hypothesis that active DNA demethylation plays an essential role in retinal cell fate specification and development [31,57]. Supporting this hypothesis, alterations to retinal development, including improper specification of the eye-field are observed in Tet3 deficient *Xenopus* [58] or improper photoreceptor development in both Dnmt1/Dnmt3a/Dnmt3b conditional mutant mice [59] and Tet2/Tet3 mutant zebrafish [60], have been observed. Mouse models removing all TET enzymes exhibit early lethality during gastrulation [61], precluding studies examining retinal development. Therefore, to fully understand the significance of DNA demethylation and 5hmC on retinal cell fate specification and development in the absence of early eye-field patterning or systemic phenotypes, conditional mouse models for Tet1, Tet2, and Tet3 are required.

In this study, we utilize an allelic series of Tet enzyme conditional mouse mutants by removing the Tet enzymes within RPCs to determine the significance of active DNA demethylation and 5hmC for retinal development. Our studies indicate that most single and double Tet mutant combinations result in normal retinal morphology and visual function. However, Tet2/3 double and Tet tcKO retinas display abnormal retinal morphology, lack visual function, and display deficiencies in photoreceptor fate specification. Inhibition of the TET enzymes disrupts DNA demethylation and 5hmC production, preventing photoreceptors from adopting a rod fate and instead increasing cone fate specification. Comprehensive transcriptional and single-base resolution 5mC and 5hmC analyses indicate substantial changes in the retinal transcriptome resulting from altered 5mC and 5hmC marks across the genome when the TET enzymes are removed. These data indicate a functional role for DNA demethylation and 5hmC for activation of critical GRNs that regulate rod fate choice. In combination, our work establishes active DNA demethylation as an initial regulator of rod fate choice, required for NRL and NR2E3 expression and the establishment of photoreceptor GRNs.

## Results

### Differential transcript enrichment of Tet transcripts within the developing retina

The cyclical process of DNA methylation/demethylation is regulated by numerous enzymes that facilitate cytosine modifications and DNA repair (Fig 1A). Single-cell RNA-sequencing (RNA-seq) of mouse [14] and human [62] retinal development identified numerous components of the DNA methylation/demethylation pathways as differentially enriched across retinal development, including transcripts encoding the DNA methyltransferases (DNMT1, DNMT3A, DNMT3B) and the TET methylcytosine dioxygenases (TET1, TET2, and TET3; Fig 1B). The DNMTs are required for proper photoreceptor development [63–64]; however, the significance of 5hmC and TET-mediated DNA demethylation for retinal development and cell fate specification remains unclear [31,60,65,66]. Tet1, Tet2, and Tet3 show differential transcript enrichment levels across retinal cell types, displaying enrichment within the photoreceptors, photoreceptor precursors, and bipolar cells (Fig 1B).

### TET enzymes are required for retinal morphology, function, and cell fate specification

5hmC modifications are prevalent within the developing nervous system [67,68] and DNA demethylation is required for glial fate specification of embryonic stem cells [69]. To determine if DNA demethylation and 5hmC deposition function similarly in the developing retina, we utilized conditional mouse knockout models targeting the TET enzymes, the key drivers of 5hmC. We conditionally deleted the TET enzymes (Tet1^*loxp/loxp*^; Tet2^*loxp/loxp*^; Tet3^*loxp/loxp*^) within developing mouse RPCs using the Tg(Chx10-EGFP/cre,-ALPP)2Clc/J (*Chx10*::Cre-GFP) transgenic line [70]. To address mosaicism of the Chx10-Cre-GFP transgene [70], we implemented a breeding strategy similar to our previous studies [14] involving Cre-positive in-crosses to ensure two functional copies of the Cre transgene. Removal of the TET enzymes within RPCs allows passive, DNA replication-dependent DNA demethylation to persist; however, TET-mediated active and passive DNA demethylation are inhibited. We first validated the efficacy of our Tet loss-of-function strategy and inhibition of 5hmC using bisulfite-assisted APOBEC-coupled epigenetic sequencing (bACE-seq). Shallow sequencing of bACE-seq libraries in postnatal day 21 (P21) retinas from Cre-negative (Cre−), Tet1^*loxp*/^+; Tet2^*loxp*/^+; and Tet3^*loxp*/^+ triple heterozygous Chx10-Cre-(+) (tHet), and Tet1^*loxp*/loxp^; Tet2^*loxp*/loxp^; Tet3^*loxp*/loxp^ Chx10-Cre(+) triple mutants (Tet tcKO) indicated a Tet dosage-dependent decrease in 5hmC across genotypes (Fig 1C; S1 Data).

The phenotypic effect of TET enzyme loss-of-function and reduced levels of 5hmC was first characterized on an allelic series of TET mutants using morphological analysis of H&E-stained retinal cross-sections from 3-week-old mice (postnatal day 21—P21). We observed that the TET enzymes are individually dispensable for retinal development (Figs 1D and S1A; S2 Data). Morphological characterizations of Tet1/2 and Tet1/3 double mutants were also indistinguishable from Cre− and tHet controls (Fig 1D and 1E; S1 Data). However, RPC-specific deletion of Tet2/3 or all three TET enzymes (Tet tcKO) resulted in abnormal retinal development, characterized by a significant attenuation of photoreceptor outer segment elongation, disruption of the outer plexiform layer (OPL) and a decrease in retinal thickness (Figs 1D, 1E, and S1A; S1 and S2 Data). The decrease in retinal thickness occurs across both the outer and inner nuclear layers (ONL and INL) (Figs 1D–1H and S1A; S1 and S2 Data). Quantification of the number of rows of photoreceptor nuclei suggests a loss of photoreceptor number or developmental disorganization in Tet tcKO retinas (S5B Fig; S3 Data). Morphological characterizations of retinas at 6 weeks showed a continued thinning of retinas and persistent lack of photoreceptor outer segment morphogenesis (Figs 1H, S1B, and S1C; S1 and S2 Data). Interestingly, the Tet2/3 cKO retinas, and particularly the INL, were noticeably thinner than retinas from all other genotypes. This may be the result of the tortuous OPL layer observed in the Tet tcKO retinas resulting in increased thickness of the INL and whole retina compared to Tet 2/3 cKO retinas.

Six-week-old mice underwent electroretinograms (ERGs) to determine the consequence of TET enzyme loss-of-function on visual function. Consistent with the observed morphological disruptions (S1B and S1C Fig; S2 Data), we identified a significant attenuation of visual function in Tet2/3 cKO and Tet tcKO mice. We observed a reduction in the dark-adapted A-wave, dark-adapted B-wave, and light-adapted B-wave in both Tet2/3 cKO and Tet tcKO animals compared to heterozygous controls (Fig 1I–1L; S1 Data), indicative of disrupted rod- and cone-photoreceptor responses to light stimuli and a failure to transduce light signals from photoreceptors to bipolar cells. The combined morphological and functional results indicate the significance of Tet2 and Tet3 enzymes - and potential redundant functions - in retinal development, as neither Tet1/2 or Tet1/3 double mutant retinas displayed gross morphological and Tet1/2 cKO mice display normal visual function.

**Fig 2:**
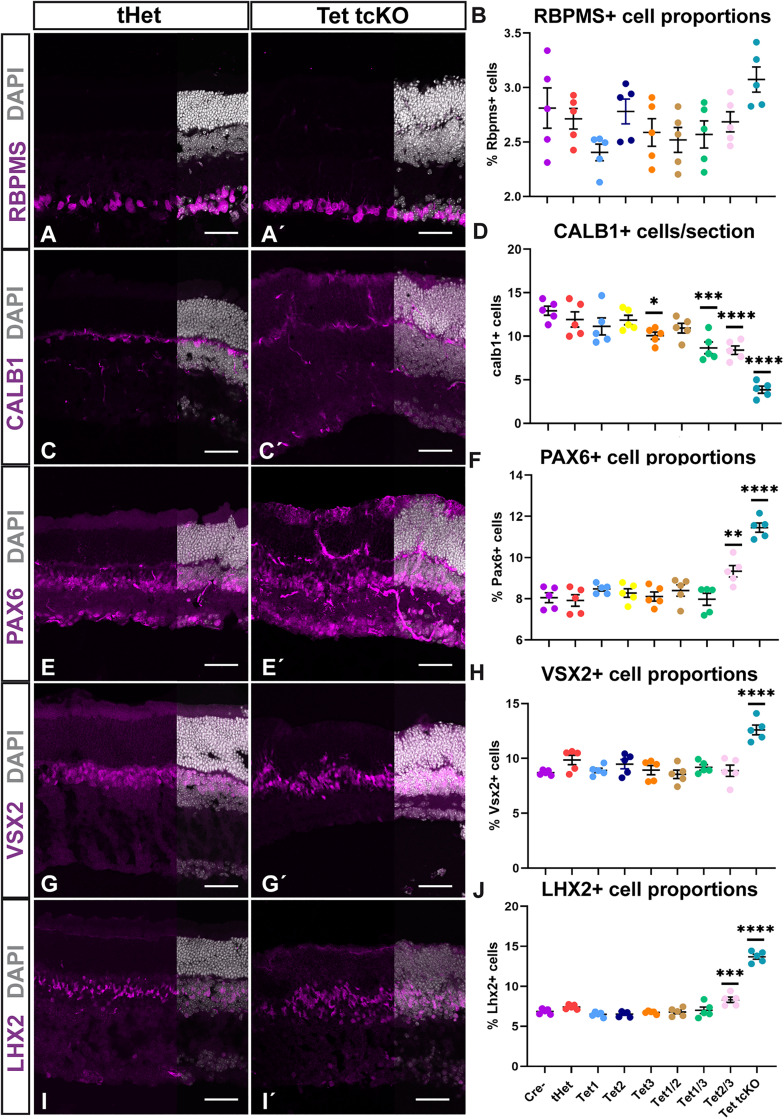
Cell fate proportions are altered in TET enzyme conditional mutant retinas. **(A, A′)** Immunohistochemistry for retinal ganglion cells (RBPMS), **(B)** Graph showing cell counts of RBPMS+ cell proportions across genotypes. **(C, C′)** Immunohistochemistry for horizontal cells (CALB1). **(D)** Graph showing cell counts of CALB1 + cell proportions across genotypes. **(E, E′)** Immunohistochemistry for amacrine cells (PAX6). **(F)** Graph showing cell counts of PAX6+ cell proportions across genotypes. **(G, G′)** Immunohistochemistry for bipolar cells (VSX2). **(H)** Graph showing cell counts of VSX2+ cell proportions across genotypes. **(I, I′)** Immunohistochemistry for Müller glia cells (LHX2). **(J)** Graph showing cell counts of LHX2+ cell proportions across genotypes. Results display the mean + SEM for *n* = 5 for each genotype. Statistics are the result of Ordinary One-Way ANOVA, followed by a Dunnett’s multiple comparisons test compared to Cre− controls. * *p* < 0.05; ** *p* < 0.01; *** *p* < 0.001; **** *p* < 0.0001. Scale bars: 100 µm. Data files for graphs available in S4 Data.

To assess the cause of reduced visual function in Tet2/3 cKO and Tet tcKO mutant retinas, we first characterized the specification of retinal cell fates using immunohistochemistry. We determined that the localization and distribution of cell type proportions across the allelic series of TET mutant retinas for retinal ganglion cells (RGCs), horizontal cells, amacrine cells, bipolar cells, and Müller glia using the cell type markers RBPMS, CALB1, PAX6, VSX2, and LHX2, respectively (Figs 2 and S2). In general, the localization of all cell types appeared normal, as cell type markers stratified to known nuclear layers. Assessments of cell type proportions across all cell type classes indicated a loss of Calb1+ horizontal cells but an increase in Pax6+ amacrine cells, Vsx2+ bipolar cells, and Lhx2+ Müller glia (Figs 2 and S2A; S4 and S5 Data). RGC numbers across the allelic series of TET mutant retinas did not result in any significant differences across genotypes. Examination of Müller glia morphology was also assessed through localization of glutamine synthetase (GS), indicating that Müller glia were present, albeit with disorganized morphology in Tet tcKO retinas (S4A Fig). Despite this disorganization, we did not observe glial reactivity as assessed by GFAP in either P21 or 6-week-old retinas (S4A Fig). While LHX2 and GS expression suggest specification of Müller glia, we also observed strong colocalization of PAX6, LHX2, and VSX2 within presumptive glial cells (S3A–S3D Fig). While all these markers are expressed in mature Müller glial cells [71–74], both PAX6 and VSX2 normally display reduced expression in glia compared to that in amacrine and bipolar cells, respectively (S3 Fig). However, in Tet tcKO retinas, the prominent co-localization of LHX2, PAX6, and VSX2 is reminiscent of transcription factor expression within RPCs, potentially resulting from a failure to complete glial differentiation and partially accounting for the observed increases in PAX6+ amacrines and VSX2+ bipolar cells in Tet tcKO retinas. The co-localization of PAX6 and VSX2 in presumptive Müller glia was confirmed by cell counts showing a significant increase in the number of PAX6/VSX2 double-positive cells (S3E Fig; S6 Data). To assess if increases in PAX6+ amacrine cells and VSX2+ bipolar cells were the result of increased Müller glia expression of PAX6 and VSX2, we also quantified proportions of TFAP2A+ amacrine and OTX2 bipolar cells. We confirmed our observations of small but significant increases in the proportions of TFAP2A+ amacrine cells (S2B and S2C Fig; S5 Data) and OTX2+ bipolar cells (S2D and S2E Fig; S5 Data). Altogether, we observed subtle yet significant changes in the specification of retinal cell fates when TET enzyme expression is lost.

To determine the potential of cell death influencing the observed changes in retinal cell fate proportions, we assessed the quantity and localization of microglia as a proxy for microglial activation in response to cell death [75]. We observe both an increase in microglial number and altered laminar distribution of microglia within nuclear and plexiform layers (Fig S4B–S4E; S7 Data). When examining the distribution of microglia within individual nuclear layers, we observe an increase of microglia in all 3 nuclear layers in Tet tcKO retinas compared with tHet controls (S4B–S4F Fig; S7 Data). To confirm these results, we also performed a TUNEL assay for apoptosis [76–77] (S4G Fig). We observed a significant increase in the mean TUNEL fluorescence in Tet tcKO retinas (S4G and S4H Fig; S7 Data). These results support previous observations that hypermethylation in photoreceptors increases cell death [77]. Therefore, we suggest that cell death contributes to a portion of the observed differences in retinal thickness and cell fate proportions in Tet2/3 and Tet tcKO retinas compared to controls.

**Fig 3:**
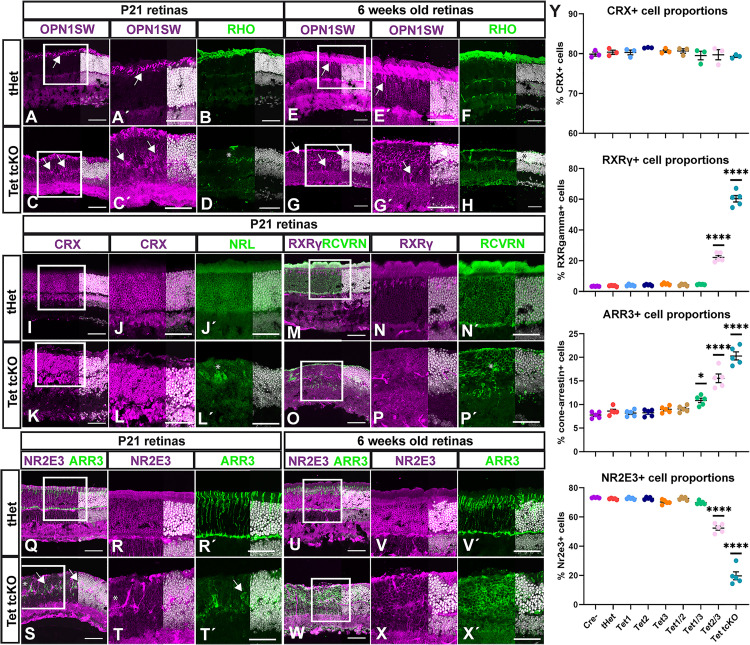
Photoreceptor fate proportions are biased towards cone photoreceptors in TET mutant retinas. **(A-H)** Immunohistochemistry for S-opsin (OPN1SW) and rhodopsin (RHO) comparing tHet and Tet tcKO retinas at P21 and 6 weeks. **(I–L′)** Immunohistochemistry for CRX and NRL comparing tHet and Tet tcKO retinas at P21. **(M–P′)** Immunohistochemistry for RXRγ and RCVRN comparing tHet and Tet tcKO retinas at P21. **(Q–X′)** Immunohistochemistry for NR2E3 and ARR3 comparing tHet and Tet tcKO retinas at P21 and 6 weeks. **(Y)** Graphs showing cell counts of CRX+, RXRγ+, ARR3+, and NR2E3+ cell proportions across genotypes. Results display the mean + SEM for *n* = 5 (*n* = 3 in the case of CRX) for each genotype. Statistics are the result of Ordinary One-Way ANOVA, followed by a Dunnett’s multiple comparisons test. * *p* < 0.05; **** *p* < 0.0001. Scale bars: 100 µm. Data files for graphs available in S8 Data.

Our ERG results indicate the lack of a functional photoreceptor (A-wave) response, potentially through failure of proper photoreceptor differentiation or function in Tet2/3 and Tet tcKO retinas. To further investigate the mechanisms by which photoreceptor dysfunction is occurring, we assessed the expression and localization of known phototransduction-related proteins. Consistent with the observed alterations in outer segment development in histological analyses (Fig 1D; S1 Data), we identified attenuated expression and localization of the opsin proteins in both P21 and 6 weeks old retinas (Fig 3A–3H). Staining for the short-wavelength cone opsin (OPN1SW) revealed maintained but mislocalized OPN1SW expression (arrows in Fig 3A, 3A′, 3C, 3C′, 3E, 3E′, 3G, and 3G′). While OPN1SW staining was localized to presumptive cone outer segments in Tet tcKO retinas, a significant fraction of OPN1SW protein also mislocalized to the ONL (arrows in Fig 3C, 3C′, 3G, and 3G′). The rod photopigment Rhodopsin (Fig 3B, 3D, 3F, and 3H) showed a near complete loss in Tet tcKO retinas (asterisks in Fig 3D and 3H) that accounts for the reduction in scotopic A-wave observed in this genotype (Figs 1I and 1L; S1 Data).

We next performed staining for CRX, a marker of both cone- and rod-photoreceptors, and observed no difference in the proportion of photoreceptors across genotypes (Figs 3I–3L, 3Y, S5A, and S5C; S8 Data). However, we observed an increase in the proportion of cells that expressed cone transcription factor RXRγ (RXRγ; Figs 3M–3P, 3Y, and S5A; S8 Data) and the cone-arrestin protein (ARR3; Figs 3Q–3T′, 3Y, and S5A; S8 Data) indicative of an increased specification of cone-photoreceptors. This increase in cone-photoreceptors was also observed at 6 weeks (Fig 3U–3X′). Consistent with OPN1SW expression patterns, ARR3 localization was disrupted in Tet mutant retinas (Figs 3 and S5A). We observed ARR3 and cone-photoreceptor nuclei localization to basal layers of the ONL (arrows in Figs 3S, 3T′, and S5D); a pattern maintained in retinas from 6-week-old Tet tcKO animals (Fig 3W–3X′). Conversely, the number of cells expressing master rod-photoreceptor transcription factors NRL [16–18,78] (Fig 3J′, 3L′) or NR2E3 [79–81] (Fig 3Q–3T, 3Y, and S5A; S8 Data) were dramatically reduced in P21 Tet2/3 and Tet tcKO retinas. Altered rod-photoreceptor fate marker expression was also observed in Tet tcKO retinas at 6 weeks of age (Fig 3F, 3H, 3M, 3N′, 3O, 3P′, 3U, 3V, 3W, and 3X), the time point used for functional analyses. Our immunohistochemical findings, together with the loss of 5hmC and the enriched expression of TET enzymes in photoreceptor precursors and photoreceptor cells during mouse retinal development (Fig 1B and 1C) underscore the critical role of TET enzymes and 5hmC in promoting rod-photoreceptor specification. The loss of NRL and NR2E3 but maintained CRX expression indicates that the TET enzymes function upstream of rod fate commitment within specified, CRX+ photoreceptor precursor cells.

Loss-of-function mutations in NRL or NR2E3 result in failure to inhibit cone-photoreceptor fate in presumptive rod-photoreceptors and manifests as enhanced S-cone syndrome in both humans and mice [16–18,78,79,81]. In our Tet2/3 and Tet tcKO retinas, we observe loss of both NRL and NR2E3 expression, an increased proportion of RXRγ+ cones, but a near complete absence of the photoreceptor-mediated A-wave even at the highest light intensities (Fig 1I–1L; S1 Data). Photopic B-wave responses, indicative of cone-bipolar cell responses, in Tet2/3 and Tet tcKO retinas are detectable, albeit greatly diminished (Fig 1K; S1 Data). To address this discrepancy of increased cone numbers but decreased second-order cone-mediated responses, we assessed the presence of synaptic connections and synaptic sub-lamina using the presynaptic photoreceptor ribbon synapse marker Bassoon [82] (CTBP2) or inner plexiform layer (IPL) sub-lamina marker Calretinin [83] (CALB2). We observe that Bassoon expression is present but reduced within the inner and outer plexiform layers (IPL and OPL) in both P21 and 6-week-old Tet tcKO retinas (S6A Fig). Calretinin staining, which labels the striata of the IPL, is disrupted in both P21 and 6-week-old Tet tcKO retinas, with striata becoming undefined in 6-week-old Tet tcKO retinas (S6B Fig). Counts of OPL Bassoon+ puncta indicate a significant decrease in the number of ribbon synapses between control and Tet tcKO retinas at P21, with an age-dependent decrease from 3 to 6 weeks (S6C Fig; S9 Data). Furthermore, colocalization of Bassoon with ARR3 staining was readily observed in cone pedicles of control retinas. We were unable to localize significant co-staining of Bassoon and ARR3 in cone pedicles of Tet tcKO retinas (S6D Fig). Our combined results indicate that disruption of retinal cell fate specification, phototransduction protein localization, and alterations to synaptic protein localization combine to cause the reduced visual function in Tet2/3 and Tet tcKO retinas.

Unlike other retinal cell types, rod-photoreceptors of nocturnal animals show an inverted nuclear structure, comprised by central heterochromatin domains with euchromatin residing peripherally [84,85]. The number of chromocenters in rod-photoreceptors decline from several chromocenters to 1–2 centralized chromocenters in mature rods [84,86–88]. To assess the degree to which photoreceptor nuclear structure was more rod- or cone-like, we assessed the number of DNA chromocenters in photoreceptor nuclei (S7 Fig; S10 Data) [88]. Consistent with a more cone-like fate, we observed alterations in the nuclear morphology of Tet tcKO retinas (S7 Fig; S10 Data). These results all support a more cone-like nuclear structure and a fate switch from rod- to cone-photoreceptors in Tet tcKO retinas.

### TET enzymes modulate retinal neurogenesis and timing of cone fate specification

During retinal development, photoreceptors are biased to differentiate as a cone ‘default’ state within early retinal development [78,89]. As development progresses, expression of Prdm1 and Nrl bias photoreceptor precursors to promote rod GRNs, including expression of Nr2e3 [17,18,78,80,90]. We observed an increase in the proportion of cone-photoreceptors (RXRγ+ cells) at the expense of rod-photoreceptors (NRL+ or NR2E3+cells) in Tet tcKO retinas. Two plausible scenarios may occur by which enhanced cone-photoreceptor fate is specified in Tet tcKO retinas: (1) TET enzyme loss-of-function results in early cell cycle exit of RPCs during an early ‘competence’ state that promotes cone specification over rod fate; or (2) Photoreceptor fate is initiated properly, but TET enzyme loss-of-function reduces the expression of rod-promoting GRNs through inhibition of NRL and NR2E3 transcription factor expression, thereby adopting a cone fate during periods of rod genesis.

**Fig 4:**
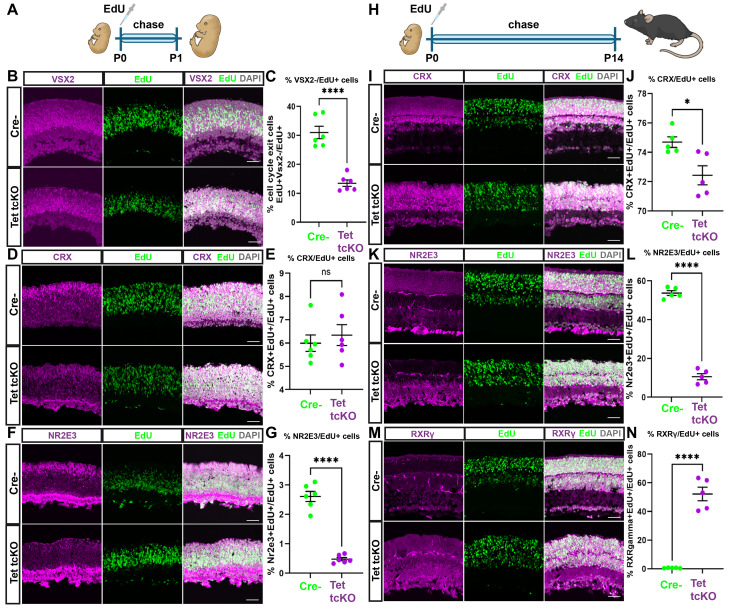
Loss of TET enzymes in RPCs alters retinal neurogenesis and extends the birth window of cone-photoreceptors. **(A, H)** Summary schemas showing the timeline of P0–P1 and P0–P14 EdU experiments. **(B, D, F, I, K, M)** Immunohistochemistry showing the labeling of progenitor cells (CHX10+), photoreceptors (CRX+), rod-photoreceptors (NR2E3+), and cone-photoreceptors (RXRγ+) in Cre− and Tet1/2/3 cKO retinas at P1 or P14 after EdU injection at P0. **(C, E, G, J, L, N)** Graphs showing proportion of cells exiting the cell cycle (CHX10+/EdU+), proportions of photoreceptors (CRX+/EdU+), proportions of rod-photoreceptors (NR2E3+/EdU+) and proportions of rod-photoreceptors (RXRγ+/EdU+). Results display the mean + SEM for *n* = 6 (P0–P1) or *n* = 5 (P0–P14) for each genotype. Statistics are the result of a two-tailed Unpaired *t* test; ns: nonsignificant; * *p* < 0.05, **** *p* < 0.0001. Scale bars: 100 µm. Data files for graphs available in S11 Data. Fig 4A and 4H were created using icons from NIH BioArt (https://bioart.niaid.nih.gov).

To begin to distinguish between these two scenarios, we performed EdU injections at P0, during the peak of rod genesis, in Cre− and Tet tcKO animals to lineage-trace RPCs (Fig 4). We first utilized a P0 to P1 EdU pulse-chase (Fig 4A) to assess the effect of TET enzyme loss-of-function on cell cycle exit. P0, EdU-labeled RPCs that exited the cell cycle were identified by the lack of co-staining of VSX2 within EdU-labeled nuclei [91–93]. We observed a decrease in the proportion of P0-P1 EdU+ cells that lack VSX2 co-expression in Tet tcKO retinas, indicating reduced neurogenesis and a maintenance of RPC proliferation (Fig 4B and 4C; S11 Data). However, the total number of VSX2+ RPCs and EdU+ cells in TET tcKO were not statistically different from controls (S8A Fig; S12 Data). Additionally, we observed a significant decrease in the proportion (PH3+/DAPI nuclei) and total number of PH3+ cells (S8B–S8D Fig, S12 Data) in Tet tcKO retinas. These data suggest that while numbers of RPCs are properly maintained, the loss of TET enzymes results in decreased mitotic divisions and reduced neurogenic capacity of postnatal RPCs during peak developmental windows of rod fate specification.

Despite observing a decrease in the neurogenic potential of P0-P1 RPCs, co-labeling of EdU+ cells with photoreceptor markers indicates that proper numbers of photoreceptors were specified during the EdU labeling period (CRX+/EdU+; Fig 4D and 4E; S11 Data). Additionally, the total number of photoreceptors in P1 Tet tcKO mutant and Cre− control retinas were similar (CRX+; S8E Fig; S12 Data). However, we did observe a reduction in the number of EdU+ cells co-labeled with NR2E3 in Tet tcKO retinas, both as a proportion of total EdU+ cells (NR2E3+/EdU+) and total number of NR2E3+ cells within the Tet tcKO retinas at P1 (Figs 4F, 4G, and S8F; S11 and S12 Data). Altogether, these results indicate that the loss of rod-photoreceptors observed in Tet tcKO retinas occurs at the peak of rod-photoreceptor specification and that postnatal RPCs display reduced neurogenic potential.

We next utilized a P0 pulse of EdU chased until P14 (Fig 4H) to determine the timing of rod- and cone-photoreceptor specification. Using CRX as the common marker of cone- and rod-photoreceptors, we observed a small but significant difference in the number of CRX+ cells generated during the P0–P14 birth window (~2% decrease in the proportion of CRX+ photoreceptor cells). Additionally, the total number of CRX+ cells in Tet tcKO retinas compared to controls was also slightly reduced (Fig 4I, 4J, and S8G; S11 and S12 Data). However, when staining for the rod-photoreceptor marker NR2E3, we show a significant decrease in the proportion of NR2E3+ rod-photoreceptors specified from P0 EdU-labeled RPCs (NR2E3+/EdU+), as well as in the total number of NR2E3+ rod-photoreceptors produced in Tet tcKO retinas (Figs 4K, 4L, and S8H; S11 and S12 Data).

Consistent with the embryonic specification of cone-photoreceptors in mice [2], we observed few P0 EdU-labeled RPCs differentiating as RXRγ+ cone-photoreceptors in Cre− control mice (less than 1%). However, EdU+/RXRγ+ co-labeled cells were detected throughout the ONL in Tet tcKO retinas (~52% of EdU+ cells; Fig 4M and 4N; S11 Data). We observed a significant increase in the number of total RXRγ+ cones (Figs 4M and S8I; S11 and S12 Data), results similar to P21 immunohistological results (Fig 3). Comparisons of NR2E3/EdU double-positive cells in controls with RXRγ/EdU double-positive cells in Tet tcKO retinas reveal that similar proportions of photoreceptors are generated in Tet tcKO and control retinas between P0 and P14 (Fig 4K–4N; S11 Data). These findings suggest that the loss of TET enzymes biases immature photoreceptors in the postnatal retina toward adopting a cone rather than a rod-photoreceptor fate, favoring the hypothesis that TET enzymes function within specified photoreceptors to promote rod fate specification, thereby preventing a cone default state. Deletion of the TET enzymes within RPCs and all resulting progeny prevents rod GRNs from being activated, including loss of NRL and NR2E3 expression. Similar to both NR2E3 and NRL mutant retinas, Tet tcKO retinas display an increase in cone-photoreceptors at the expense of rod-photoreceptors.

### Molecular characterizations of TET enzyme loss-of-function within the mature retina

#### TET enzymes regulate retinal GRNs for determination of photoreceptor fates.

Our immunohistochemical characterizations of Tet tcKO retinas led us to hypothesize that TET enzymes are required for promoting rod-photoreceptor fate specification. RNA-seq experiments on both P21 bulk tissue and isolated single nuclei were performed to further characterize the changes in transcript expression resulting from TET enzyme loss-of-function. In bulk RNAseq experiments, we isolated both RNA and DNA from retinas pooled from two animals for Cre negative (Cre−), Tet1^*loxp/+*^, Tet2^*loxp/+*^, Tet3^loxp/+^ triple heterozygous *Chx10*::Cre-GFP positive (tHet), and Tet tcKO retinas. RNA was purified for RNA-seq while matched DNA samples were isolated for 5mC and 5hmC profiling (see below; S9A Fig).

Efficiency of our triple conditional knockout approach was assessed by determining the number of reads mapping to TET transcripts, the percentage of TET transcripts overlapping floxed exons, and the canonical splicing efficiency in Tet tcKO retinas compared to controls (Fig S9B–S9E; S13 Data). We observe a Cre-dependent dosage decrease in the percentage of properly spliced-in reads of Tet floxed exons (spliced in reads over the sum of both spliced in and spliced out reads) across Cre−, tHet, and Tet tcKO retinas (S9E Fig; S13 Data).

**Fig 5:**
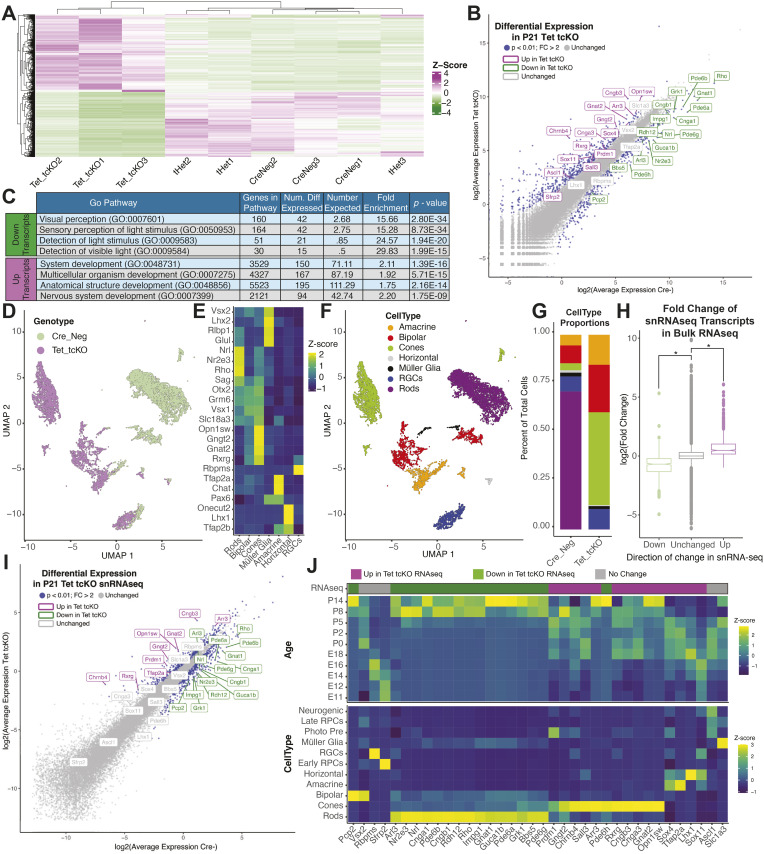
Tet loss-of-function promotes cone photoreceptors GRNs at the expense of rod photoreceptors. **(A)** Heatmap of differential transcripts across bulk P21 retinal RNAseq replicates. **(B)** RNA transcript expression in Cre− and Tet tcKO retinas, colored by differential expression significance and direction of change. **(C)** Gene Ontology (GO) analysis of differentially expressed transcripts indicating enriched Biological Pathways of Up- and Down-regulated transcripts. **(D)** UMAP dimension reduction of retinal neurons and glia from snRNAseq on P21 Cre− and Tet tcKO retinas, with cells colored by genotype. **(E)** Heatmap showing relative expression enrichment of cell type markers within snRNAseq annotated cell types. **(F)** UMAP dimension reduction of retinal neurons and glia from snRNAseq on P21 Cre− and Tet tcKO retinas colored by annotated cell type. **(G)** Proportions of annotated cell types by genotype. **(H)** Boxplots displaying the relative fold change of snRNAseq differentially expressed transcripts in bulk RNAseq experiments. Asterisks represent *p*-values from Wilcoxon Rank Sum statistical comparisons; * *p* < 2.2e−16 **(I)** Pseudo-bulked, average transcript expression of snRNAseq experiments from Tet tcKO and Cre−. Transcripts are colored by differential expression significance and direction of change. **(J)** Developmental and cell type expression enrichment of differentially expressed transcripts from Tet tcKO RNAseq experiments in the retinal development dataset [14]. Data files for graphs available in S14 Data.

We observed differential expression of 1,102 transcripts in Tet tcKO retinas compared to Cre− samples (Fig 5A; 627 up-regulated and 475 down-regulated transcripts, log2(Fold Change) >1, False Discovery Rate (FDR) < 0.01). 691 (62.70%) transcripts displayed differential expression in both Tet tcKO versus Cre− and Tet tcKO versus tHet pairwise comparisons. All 691 transcripts displaying differential expression in both Tet tcKO pairwise analyses displayed a congruent direction of transcript expression changes across Cre− versus Tet tcKO and tHet versus Tet tcKO pairwise comparisons (331 down-regulated transcripts and 360 up-regulated transcripts).

Genes displaying decreased transcript expression include rod-photoreceptor transcription factors, *NRL* (log2 fold change (log2FC) = −2.30; FDR = 1.03e−15) and *NR2E3* (log2FC = −2.13; FDR = 2.70e−12) and rod phototransduction genes *PDE6A* (log2FC = −3.15; FDR = 1.96e−9), *GNAT1* (log2FC = −2.93; FDR = 1.59e−12), and *RHO* (log2FC = −2.98; FDR = 4.14e−6). Conversely, up-regulated transcripts included cone transcription factor *RXRG* (log2FC = 2.49; FDR = 1.07−e13) and cone phototransduction genes *GNGT2* (log2FC = 2.13; FDR = 3.94e−9)*, *GNAT2** (log2FC = 1.94; FDR = 2.63e−14), and *OPN1SW* (log2FC = 1.62; FDR = 1.44e−8). Importantly, we did not observe statistically significant changes in gene expression for photoreceptor transcription factor *CRX*, or cell type-specific markers *RPBMS*, *TFAP2A*, *LHX1*, *VSX2*, or *SLC1A3*, markers of RGCs, amacrine cells, horizontal cells, bipolar cells, or Müller glia, respectively (Fig 5B; S2 Table). Gene Ontology (GO) pathway analyses of differentially expressed transcripts indicated an enrichment of down-regulated transcripts in biological pathways related to visual perception and detection of light (Fig 5C; S3 Table). Conversely, up-regulated transcripts were enriched in pathways related to regulation of development, including nervous system development (Fig 5C; S3 Table).

Differential expression analysis of Cre− and tHet samples revealed minor differences in RNA transcript abundance across genotypes (37 up-regulated transcripts, 2 down-regulated transcripts, log2(Fold Change) > 1, FDR < 0.01; S9F Fig; S2 Table), indicating minor differences in Cre− and tHet control models. This reduced level of expression changes is consistent with smaller changes in global 5hmC levels observed in the tHet mice (Fig 1C; S1 Data).

To further confirm cellular identity changes observed in immunohistochemical data, we performed single-nucleus RNA-sequencing (snRNA-seq) of P21 retinas. For both Tet tcKO and Cre− control samples, nuclei from two independent pools of nuclear dissociations (≥2 animals, ≥4 retinas each) were utilized for input into Particle-templated instant partition sequencing (PIPseq) snRNA-seq reactions [94]. Initial snRNA-seq processing and dimension reduction revealed largely distinct clustering of Tet tcKO nuclei from Cre− controls (Figs 5D and S9G–S9I). Cell type calls were performed using enrichment of canonical cell type marker genes within clusters (Fig 5E and 5F; [14]), determining an increase in cone-photoreceptors in Tet tcKOs compared to the Cre− control sample (Fig 5G). Differential expression analysis (Monocle3 fit_models; *q*-value < 1e−20) across all cells by genotype revealed 518 up- and 523 down-regulated transcripts in Tet tcKO cells compared with Cre− controls (S4 Table). Comparisons of differentially expressed transcripts in either snRNA-seq or bulk RNA-seq samples revealed consistent patterns of transcript expression changes between RNA-seq modalities (Fig 5H and 5I; S14 Data). Examination of the cell type and temporal enrichment of differentially expressed transcripts from Tet tcKO retinas across normal retinal development [14] confirmed an up-regulation of cone-enriched transcripts and loss of rod transcripts (Fig 5J, bottom). As the peak birth windows of cone photoreceptors precedes that of rod photoreceptors [2,14], we observed that cone-enriched, up-regulated transcripts in Tet tcKO retinas are normally expressed earlier than down-regulated transcripts during retinal development (Fig 5J, top).

To gain further insight into the cellular enrichment of differentially expressed transcripts from transcript profiling experiments, we examined the cellular expression of transcripts as ‘gene modules’ in the single-cell atlas of the developing mouse [14]. Consistent with our immunohistochemical data highlighting changes in photoreceptor fate specification, down-regulated transcripts in Tet tcKO retinas display preferential expression within rod-photoreceptors while up-regulated transcripts are preferentially expressed within cone-photoreceptors (S10A–S10E Fig). Furthermore, to assess the degree of cone maturation in Tet tcKO retinas, we identified transcripts from our previous developmental single-cell atlas [14] that are specifically expressed during cone specification or those expressed highly within mature cones (S10F Fig). We observe that many transcripts expressed within differentiating cones are up-regulated in Tet tcKO retinas in both our bulk and snRNA-seq experiments. While many mature cone transcripts are up-regulated (**Opn1sw*, *Arr3*, *Gnat2**), we observed a significantly decreased transcript abundance of mature photoreceptor transcripts (**Rcvrn*, *Cplx4*, *Prph2**) in both RNA profiling experiments and a decrease in mature, cone-specific transcripts *(Kcne2* and *Pde6h*) in bulk profiling experiments (S10G Fig; S15 Data). The significant decrease in phototransduction gene transcript expression (**Rcvrn*, *Splx4*, *Prph2**) may partially explain the lack of cone-mediated visual function. In combination with the immunohistochemical data, both our bulk RNA-seq and snRNA-seq data support a bias in photoreceptor fate specification whereby rod fate is inhibited when TET enzyme function and 5hmC are lost. Instead, photoreceptor precursors adopt an earlier developmental transcriptional program and are biased to differentiate as a cone default fate.

#### TET enzymes regulate global methylation programs during retinal development.

Our immunohistochemical and RNA profiling data highlight a requirement of the TET enzymes for rod-photoreceptor fate specification and differentiation. We next sought to quantify alterations to DNA methylation patterns that control rod-photoreceptor fate specification and retinal gene expression at base pair resolution.

**Fig 6:**
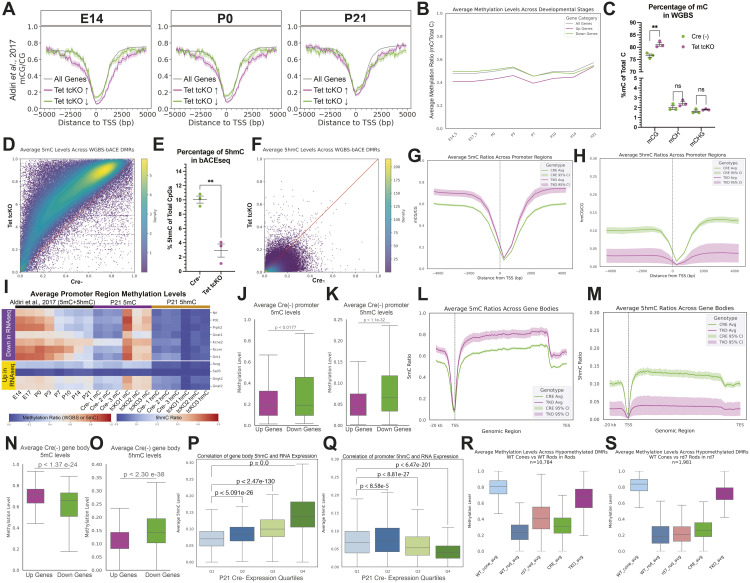
Tet tcKO results in dramatic changes in the retinal methylome. **(A)** Comparisons of the temporal WGBS methylation patterns at early (E14) mid (P0) and late (P14) timepoints of retinal development across the ± 5 kb of the TSS for up- and down-regulated transcripts from Tet tcKO RNAseq experiments. **(B)** Line graph displaying the average CpG methylation levels across retinal development of the proximal TSS for up- and down-regulated transcripts across retinal development. **(C)** Scatterplot of the average methylation levels from WGBS performed on P21 Cre− and Tet tcKO samples for mCG, mCH, and mCHG. **(D)** Density plot of DMRs for 5mC between P21 Tet tcKO and Cre− control retinas. **(E)** Scatterplot of the average 5hmC levels from bACE-seq performed on P21 Cre− and Tet tcKO samples. **(F)** Density plot of DMRs for 5hmC between P21 Tet tcKO and Cre− control retinas. **(G, H)** Line plot showing the average (G) 5mC or (H) 5hmC levels across the proximal promoter Tet tcKO and Cre (-) control retinas. **(I)** Heatmaps displaying temporal methylation patterns and 5mC and 5hmC profiles of the promoter regions in Tet tcKO and Cre− control retinas for selected, differentially expressed transcripts from RNAseq experiments. **(J, K)** Boxplots of promoter (J) 5mC and (K) 5hmC levels for genes that display differential transcript expression in RNA-seq experiments (P21 Tet tcKO compared to Cre− controls). **(L, M)** Line plots showing the average (L) 5mC or (M) 5hmC levels across gene bodies in Tet tcKO and Cre− control retinas. **(N,O)** Boxplots displaying average (N) 5mC or (O) 5hmC across gene bodies of differentially expressed transcripts in RNA-seq experiments (P21 Tet tcKO compared to Cre− controls). **(P, Q)** Boxplots displaying average 5hmC levels across the (P) gene body or (Q) promoter for all genes, binned in quartiles by transcript expression levels in P21 Cre− RNA-seq. Statistics represent results of a Wilcoxon Rank Sum Test for pairwise comparisons of Quartile 1. **(R, S)** Boxplots of average methylation profiles for differentially hypomethylated regions identified in comparisons between (R) Rods vs. Cones and (S) *rd7* Rods vs. Cones in P21 Cre− and TET tckO retinal samples and sorted cones, rods, and *rd7* rods. Statistical analyses and G represent the results of unpaired student *t* tests (C, G, L–O; ** *p* < 0.01, ns, not significant). Data files for graphs available in S17 Data.

Previous characterizations of modified cytosines implicated promoter and gene body DNA methylation status as key epigenetic marks for the regulation of transcript expression [95]. We reanalyzed a temporal series of retinal DNA methylation profiling via Whole Genome Bisulfite Sequencing (WGBS) [27] to determine the developmental dynamics of DNA methylation status and interrogated how developmental DNA methylation profiles correlate with differentially expressed transcripts in Tet tcKO retinas. As WGBS fails to distinguish between 5mC and 5hmC modifications (S11A Fig), these analyses only compare the levels of modified cytosines (5mC + 5hmC) to unmodified cytosines. We observe that during embryonic and early postnatal development (E14-P0), cone-enriched, up-regulated genes within Tet tcKO retinas display fewer modified cytosines within ±5000 nucleotides of the transcription start sites (TSS) compared to all other genes (Figs 6A, 6B, and S11B). Conversely, rod-enriched, down-regulated genes in Tet tcKO retinas display more similar methylation to non-differentially expressed genes but greater than genes displaying increased expression in Tet tcKO retinas (Figs 6A, 6B, and S11B). These differences in global DNA methylation status are reduced as development progresses (P7-P21; Figs 6A, 6B, and S11B). Sequence downstream (0 to +5,000 base-pairs (bp) of both up- and down-regulated genes at P21 display less DNA methylation than non-differentially expressed transcripts (Figs 6A and S11B), consistent with active maintenance of reduced DNA methylation to facilitate gene expression [31,66].

We next examined the consequence of Tet enzyme loss-of-function on the retinal methylome. Using the matched DNA from RNA-DNA extractions in RNA-seq experiments, we performed both whole methylome profiling (5mC + 5hmC) via WGBS and 5hmC profiling through bACE-seq [96, 97] (S11A and S11C–S11E Fig). Shallow sequencing of bACE-seq samples in Cre−, tHet, and Tet tcKO retinas indicated that ~10% of CpG sequences harbor 5hmC modifications within the P21 retina (Fig 1C; S1 Data); a number consistent with 5hmC levels in other profiled nervous system tissues [98]. Estimates of non-methylated cytosine conversion rates averaged approximately 98% (2% unmodified cytosines failed to be converted; S11C Fig; S16 Data), suggesting robust conversion of cytosine to thymine in bACE-seq reactions.

Deep sequencing of both WGBS and bACE-seq libraries followed by MLML deconvolution analyses [99] to resolve 5mC and 5hmC signatures in WGBS provided base-pair resolution of 5mC or 5hmC methylation status (S11D–S11F Fig). Differentially methylated region (DMR) analyses of WGBS and bACE-seq indicated a large-scale maintenance of cytosine modifications (5mC + 5hmC) in WGBS (191,164 DMRs with up-regulated 5mC + 5hmC and 2,672 DMRs losing 5mC + 5hmC; Fig 6C and 6D; S17 Data; S5 Table). Our bACE-seq results provide the first base-pair resolution of 5hmC within the mouse retina and identified a dramatic loss of 5hmC across the genome (592,697 DMRs losing 5hmC, 209 DMRs gaining 5hmC; Fig 6E and 6F; S6 Table) in Tet tcKO mutant retinas, consistent with the requirement of the TET enzymes for oxidizing 5mC to 5hmC in post-mitotic cells. Non-CpG methylation levels were lowly abundant and unaffected by deletion of the TET enzymes (Fig 6C; S17 Data). The low levels of non-CpG methylation in the retina are consistent with previous reports [30].

Examination of the 5mC levels across the TSS of all genes indicates a global enrichment of 5mC across the proximal promoter region in Tet tcKO retinas (Fig 6G). Conversely, bACE-seq analysis determined that global levels of 5hmC were significantly reduced across the promoter in Tet tckO retinas (Fig 6H). We next sought to understand the biological implications of changes in methylation in the Tet tcKO retinas by comparing the temporal dynamics and cell-type-specific patterns of methylation across retinal development. Combining the temporal time-series of WGBS methylation profiling [27] with our deconvolved 5mC and 5hmC profiling, we next examined how promoters of differentially expressed genes in Tet tcKO retinas are methylated. We observe that promoters of many down-regulated genes in Tet tcKO retinas display high methylation levels in early development that are lost at time points where these genes become highly expressed in the retina (P3-P7 through P21; Fig 6I). Conversely, many up-regulated gene promoters display low levels of DNA methylation that are, on average, less dynamic across development (Fig 6I, bottom). Our P21 methylation profiles observe low levels of 5mC in promoters for all differentially expressed genes in P21 Cre− retinas; however, Tet tcKO retinas display increased 5mC levels at promoter regions, occurring for both up and down-regulated genes (Fig 6I). Similarly, low levels of 5hmC are observed in P21 Cre− samples across both up- and down-regulated genes, with reduced 5hmC levels observed in Tet tcKO knockouts. These results suggest that removing the TET enzymes prevents the DNA demethylation of specific promoters during retinal development, thereby preventing the expression of a subset of genes—those observed as down-regulated in our RNA-seq. We then directly compared promoter and gene body methylation levels of up- and down-regulated genes within the wild-type (Cre−) retina. Promoter 5mC levels between up- and down-regulated transcripts were significantly different despite the mean 5mC levels being similar (Up-regulated gene promoter 5mC = 26.6% of CpGs; Down-regulated promoter 5mC = 28.17%; Fig 6J, S17 Data). Conversely, we observed lower promoter 5hmC levels in genes that are up-regulated compared to down-regulated genes (Fig 6K, S17 Data). Together, these data highlight that cone-enriched, up-regulated transcripts in Tet tcKO retinas have less promoter methylation (both 5mC and 5hmC) and therefore do not require DNA demethylation to activate transcription. Conversely, loss of the TET enzymes prevents removal of 5mC and the accumulation of 5hmC modifications at promoters of rod-promoting genes, thereby inhibiting expression of these genes.

Analysis of the methylation across gene bodies followed similar patterns to that of promoter sequences. We observed that gene body 5mC levels are increased across the gene body (TSS to transcription termination site; TTS) compared to the proximal promoter region in Cre− control retinas (Fig 6L). Deletion of the TET enzymes results in an increase of 5mC levels from ~60% to ~80% of 5mC-modified CpGs across the gene body (Fig 6L). We also observed a global decrease in 5hmC levels across the gene bodies in Tet tcKO retinas (Fig 6M). We then analyzed the P21 wild-type (Cre−) methylation levels of differentially expressed transcripts from RNA-seq of P21 Tet tcKO retinas to gain a better understanding of how gene body methylation levels affect RNA expression. We observed that, on average, down-regulated transcripts display lower 5mC and higher 5hmC gene body levels in the wildtype retina than up-regulated transcripts (Fig 6N and 6O; S17 Data), suggesting a requirement of DNA demethylation and potentially 5hmC modifications across the gene body to promote RNA expression. Loss of the TET enzymes maintains gene body 5mC levels of down-regulated genes and inhibits transcription.

Gene body 5hmC levels have previously been associated with higher levels of RNA transcription [100,101]. To assess the correlation between promoter and gene body 5hmC levels with expression, we binned all genes in RNA-seq experiments by ordered quartiles of expression levels and compared average promoter or gene body 5hmC levels. In agreement with previous reports, we observed that genes with higher levels of RNA transcript expression exhibit higher levels of gene body 5hmC (Figs 6P and S11J; S17 Data). We did not observe a robust correlation of the 5hmC promoter levels with high RNA transcript expression, although highly expressed transcripts did display lower 5hmC promoter levels on average (Figs 6Q and S11K; S16 and S17 Data).

To further examine the temporal regulation of methylation and the effect of TET enzyme loss-of-function on the regulation of retinal gene expression, we examined methylation levels of *cis*-regulatory elements (CREs). We utilized a temporal single-nucleus Assay for Transposase Accessible Chromatin (snATAC) across retinal development [102] to identify all accessible chromatin regions in retinal cells. We then determined the identity of the sites as promoters (±2,000 bp from TSS), gene body, or distal ATAC sequences (ATACseq peaks from [102] not associated with promoters or gene bodies). Using the previous characterizations of the temporal dynamics of methylation (5mC + 5hmC) [27], we identified the CREs that normally undergo DNA demethylation across retinal development (>10% reduction in methylation across development; 42,473 Distal ATAC peaks and 162 promoter sequences). Comparison of our Cre− P21 WGBS methylation patterns in enhancer and promoter sequences correlated well with more mature retina methylation profiles (P10–P21) but poorly with earlier developmental timepoints for both distal ATAC sequences and promoters (S11I and S11J Fig). This result is consistent with dramatic changes in methylation patterns of accessible regulatory elements across development as retinal cell fates are specified and cells mature. Conversely, the P21 Tet tcKO methylation patterns correlated highest with the E14.5 WGBS methylation patterns for both distal ATAC and promoter sequences (S11H and S11I Fig). Together, these results indicate that loss of the TET enzymes within the RPCs early in retinal development promotes an early developmental methylation profile and prevents dynamic changes in methylation patterns.

As we observed an increase in cone-photoreceptor cells at the expense of rod-photoreceptors, we next compared our Cre− and Tet tcKO WGBS methylation profiles to those of sorted rod- and cone-photoreceptors [30]. As we observe a dramatic decrease in NR2E3 expression in Tet tcKO retinas at both the transcript and protein level, we also compared Tet tcKO methylation profiles to methylation profiles of sorted photoreceptors from *rd7* mice, a model where the rod-promoting NR2E3 transcription factor is mutated and all photoreceptors are cone-like [30,79,80,103]. Comparisons of methylation levels across hypomethylated regions in rod-photoreceptors or *rd7* rods compared to cones identified that Cre− samples display methylation levels more similar to rods and *rd7* rods than cones (Fig 6R and 6S; S17 Data). However, Tet tcKO methylation profiles are more similar to those of cones in both comparisons, indicating maintained methylation of genomic regions in Tet tcKO retinas that normally undergo demethylation in rods (Fig 6R and 6S; S17 Data). Importantly, loss of NR2E3 in the *rd7* model displays a methylation pattern more similar to rods than cones, suggesting that methylation patterns in rods are established upstream of NR2E3 function (Fig 6R and 6S; S17 Data). Examination of the methylation status of genomic regions that are hypomethylated in cones, however, indicates that Tet tcKO retinas maintain high methylation of these sites (S11K and S11L Fig; S16 Data), suggesting that while Tet tcKO retinas display expression of numerous cone marker genes, active DNA demethylation is required to fully establish the proper cone methylome. While Tet enzyme loss of function promotes cone-photoreceptor fate, cone-photoreceptor function is likely impaired due to incomplete maturation of the cells as a consequence of altered DNA methylation.

Association of 5hmC DMRs with genomic features indicates that >30% of 5mC and 5hmC DMRs were localized to Distal Intergenic regions (S11M Fig). Distal enhancer sequences displayed the highest average change in 5hmC modifications in Tet tcKO retinas (S11N Fig; S18 Data—https://doi.org/10.6084/m9.figshare.29575223.v1). As methylation status affects transcription factor binding to DNA [104], future analysis into the significance of 5hmC deposition on enhancer sequences will determine the positive or negative effects of 5hmC on enhancer activity. However, examination of the distribution of DMRs across snATAC peaks in photoreceptor gene loci provided further support for the requirement of DNA demethylation for rod development (S12 Fig). Cone-photoreceptor loci (*Rxrg*, *Arr3*, and *Opn1sw*) showed little change in 5mC levels (purple bars) across accessible peaks (blue bars). 5hmC levels displayed larger changes with loss of the TET enzymes. Conversely, in both genetic loci of photoreceptor precursor cells (*Crx*, *Otx2*, and *Prdm1*) and rod-photoreceptors (*Nrl*, *Nr2e3*, and *Rho*), we observed large regions of DMRs that overlapped regulatory sequences that gained 5mC and lost 5hmC (S12 Fig). Further studies into the mechanisms by which the Tet enzymes are targeted to these loci to promote site-specific DNA demethylation are required.

**Fig 7:**
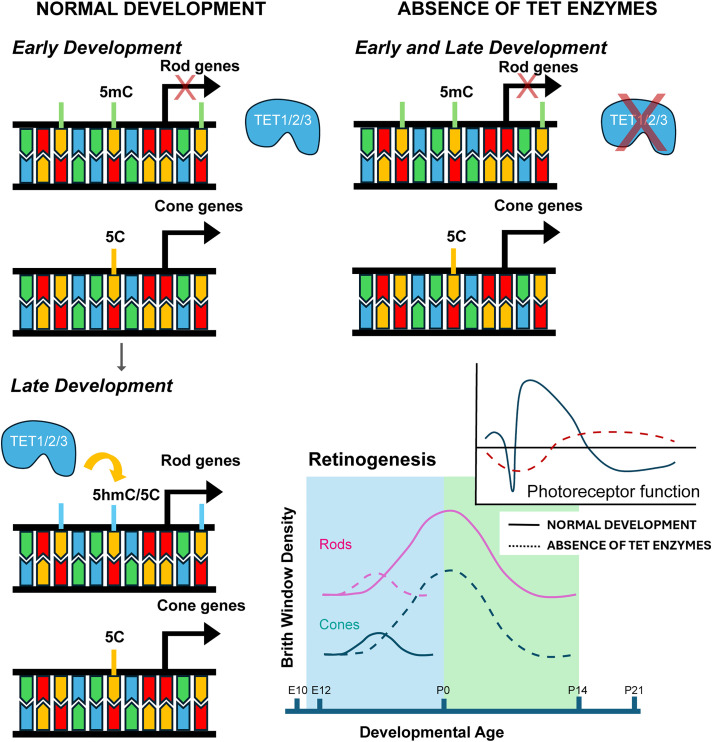
Model of the molecular mechanisms by which TET-mediated DNA demethylation regulates photoreceptor fate decisions. During early retinal development rod-photoreceptor genes (including NRL and NR2E3) are methylated. As cone-photoreceptor genes are lowly methylated, cone fate is favored. As development progresses, the TET enzymes mediate demethylation of rod-promoting genes. In the absence of TET enzymes, demethylation of NRL, NR2E3, and other genes is inhibited, preventing expression and leading to a retina where all photoreceptors are specified as cones.

Altogether, our results highlight the requirement of the TET enzymes and DNA demethylation for the proper specification of rod-photoreceptors during retinal development. We identified a novel mechanism regulating retinal cell fate specification, placing the TET enzymes upstream of rod fate choice in photoreceptor precursors. We show that expression of both NRL and NR2E3, master rod-photoreceptor transcription factors, are inhibited when DNA demethylation is impaired. Base-pair resolution profiling of both 5mC and 5hmC highlights the large-scale dynamics of TET-mediated DNA demethylation, including maintenance of 5mC modifications within the NRL and NR2E3 loci in Tet tcKO retinas. Therefore, we hypothesize a model by which cone-photoreceptor fate is promoted during early development because cone-promoting genes are not actively regulated by DNA demethylation. Rod-photoreceptor fate, however, requires TET-mediated DNA demethylation within post-mitotic photoreceptor precursors, whereby expression of NRL and NR2E3 are induced to inhibit cone fate, enabling the developmental switch from cone to rod fate specification (Fig 7).

## Discussion

Here, we describe a novel mechanism for the regulation of photoreceptor fate choice within the developing retina. Using a conditional knockout mouse model to remove the TET enzymes from developing RPCs, we show that TET enzyme-mediated DNA demethylation is required for specification of rod-photoreceptors. Removal of the TET enzymes results in a cone-rich retina, while proportions of other major retinal cell types are largely maintained. We observe a requirement of the TET enzymes within CRX+ photoreceptor precursors for the expression of master rod-photoreceptor transcription factors NRL and NR2E3. Our results, including comprehensive transcriptional profiling in conjunction with base-pair resolution profiling of 5hmC and 5mC modifications indicate that oxidation of 5mC to 5hmC is vital to retinal development and function. We observed that 5hmC is maintained across ~10% of all CpGs and that 5hmC modifications over gene bodies is correlated with gene expression levels.

Previous studies have identified that numerous promoters and gene bodies of photoreceptor genes (including NRL and NR2E3) are methylated in progenitors but exhibit low DNA methylation levels and high chromatin accessibility in mature photoreceptors [29–31]. Combined with the enriched expression of demethylation pathway components within the photoreceptor lineage (Fig 1B), this has led to the hypothesis that DNA demethylation, and therefore the TET enzymes, are vital for photoreceptor fate determination, maturation, and function. Previous studies in Tet2^−/−^;Tet3^−/−^ zebrafish show RGC differentiation deficiencies, including altered optic nerve development [60]. However, in our studies, we did not find significant differences in the number of RGCs. A recent similar study observes that conditional deletion of the TET enzymes results in a thinner optic nerve and progressive retinal degeneration, suggesting that prolonged dysregulation of DNA demethylation results in cell death [66]. This result is supported in our model by the increase in nuclear-localized microglia and TUNEL staining in TET tcKO retinas (S4B–S4H Fig; S7 Data). Zebrafish Tet2/3 mutant retinas display problems in the terminal differentiation of photoreceptors and the absence of their outer segments [60,65]. However, we observe a fate switch in photoreceptors, characterized by an increase in cone-photoreceptors at the expense of rod-photoreceptors when DNA demethylation is inhibited. This discrepancy in models may be the result of either species-specific differences in cell type composition or compensation from Tet1 as TET enzymes display partial functional redundancy [55,105,106]. In our Tet tcKOs, we also observe photoreceptor dysfunction and reduced transmission of visual signals to second-order neurons (i.e., bipolar cells). Lineage tracing analyses in Tet tcKO retinas highlight the normal specification of photoreceptor numbers but altered photoreceptor subtype fate specification. Histological and RNA-seq analyses (Figs 2–5) confirmed both lack of NRL and NR2E3 RNA transcript and protein expression, leading to enhanced specification of a cone-photoreceptor ‘default’ state.

Interestingly, we also observe small changes in numbers of mitotic divisions, neurogenic potential of RPCs, and increased proportions of INL cell types, including TFAP2A+ amacrine and OTX2+ bipolar cells in Tet tcKO retinas. One mechanism by which this may occur is through a global developmental delay as observed previously in zebrafish Tet mutants [65]. Conversely, the decreased neurogenic potential of post-natal RPCs may result in an expanded birth-window of late-born retinal cell types, including GABAergic amacrine cells, bipolar cells, and Müller glia. Alternatively, the photoreceptor cell death may cause a denominator effect, slightly increasing the proportions of INL cell types by reducing the total number of cells through loss of photoreceptors. However, we do not believe that changes in photoreceptor fate cause non-autonomous biases in specification of other retinal cell types. Previous studies of NRL and NR2E3 knockouts have shown that rod bipolar cell specification proceeds normally when rod fate is inhibited [107,108]. Furthermore, the increased proportion of INL cells or disrupted patterning of the INL in Tet tcKO retinas may result in an increased thickness of the INL compared to Tet2/3 cKO retinas (Fig 1G).

Previous studies have shown that loss of either NRL or NR2E3 leads to development of cone-dominant retinas that display decreased expression of rod-specific genes such as Rhodopsin, Recoverin or GNAT1 and increased expression of cone-specific genes including PDE6C, GNAT2, or OPN1SW. In both NRL and NR2E3 mutant mouse models, failure to fully specify rod-photoreceptors leads to retinal degeneration. In humans, disruption of either NRL or NR2E3 expression causes enhanced S-cone syndrome, characterized by supranormal blue cone function due to an increased proportion of S-cones and night blindness due to the absence of rod-photoreceptors [79,81]. This phenotype has also been described in NR2E3-null human organoids, which show the disruption of photoreceptor cell fate and maturation [109]. Expression profiling of the Tet tcKO retinas indicates similar changes in gene expression to NRL or NR2E3 loss-of-function models, leading us to propose a model whereby TET enzymes are upstream of NRL and NR2E3 for specification of rod-photoreceptors within post-mitotic photoreceptor precursor cells (Fig 7). However, the observed increase in cone-photoreceptors in Tet tcKO retinas does not lead to an increase in cone photoreceptors function like observed in enhanced S-cone syndrome. This discrepancy may result from improper establishment of synapses between cones and down-stream neurons and/or decreased expression of a subset of mature cone photoreceptor transcripts, including *Rcvrn*.

Our methylation profiling experiments (Fig 6) highlight global changes in the retinal methylome in Tet tcKO retinas. We observe the loss of 5hmC and maintained 5mC in both the NRL and NR2E3 gene loci (S12 Fig), indicating that the TET enzymes likely regulate NRL and NR2E3 expression and rod-photoreceptor GRNs. Therefore, NRL and NR2E3 deficient photoreceptor precursors adopt a cone default fate in Tet tcKO retinas, highlighted by expression of the cone transcription factor RXRγ. However, despite the increase in the number of cone-photoreceptors, the Tet tcKO cones do not display DNA methylation profiles similar to those of mature cones. We suggest that the TET enzymes are required for full maturation of cone photoreceptors and that loss of the Tet enzymes inhibits cone function and alters retinal morphology. These results are consistent with additional Tet mutant mouse and zebrafish models recently reported [65,66].

Our results highlight the requirement of active DNA demethylation within photoreceptor precursors to promote rod-photoreceptor fate specification. CRX, the cone-rod homeobox transcription factor, acts upstream of both NRL and NR2E3. CRX initiates expression in mice at E12.5 in post-mitotic specified photoreceptor precursors [110–112]. CRX binding coordinates the expression of rod- or cone-photoreceptor genes required for individual photoreceptor subtype specification [113,114]. Mutations in the CRX sequence or changes in its binding sites lead to several retinal diseases, including Leber Congenital Amaurosis, Cone-rod Dystrophies, and Retinitis Pigmentosa [115–117], as well as changes in chromatin remodeling in specific target sites [114]. We observe that CRX expression is unchanged in Tet tcKO retinas despite alterations to the methylation patterns in the *Crx* locus (S11 Fig), indicating that initial CRX-dependent photoreceptor specification is undisturbed. However, recent work has explored the consequence of DNA methylation on CRX binding to DNA, identifying altered CRX binding to the CRX consensus motif (TAATCC) by the presence of cytosine methylation modifications and DNA demethylation intermediates (5mC, 5hmC, 5fC, and 5caC) [118]. Our methylation profiling results highlight that cone-enriched, up-regulated genes in Tet tcKO retinas display reduced 5mC levels and less temporal dynamics across development (Figs 6 and S11). Conversely, the loss of TET enzyme expression prevents the demethylation of rod-enriched transcripts. Therefore, we suggest that the cone-enriched Tet tcKO retina may be a result of failure of CRX to bind and activate gene expression in rod gene loci due to altered CRX binding affinities when DNA methylation is maintained.

While we have identified plausible mechanisms for why cone fate is promoted in Tet tcKO retinas, we do not yet fully understand how the TET enzymes are targeted to specific rod-promoting gene loci to promote DNA demethylation within photoreceptor precursors or how this process is regulated in a temporal manner consistent with the temporal specification of retinal cell fates. Although TET enzymes have partially redundant functions in central nervous system development, recent evidence suggests only slight sequence preference for each TET enzyme [119]. It is more likely that the TET proteins interact with other cofactors to drive sequence and context-specific DNA demethylation. The GADD45 proteins are reported to promote demethylation by mediating TET enzyme localization to DNA [120–124]; however, the significance of the GADD45 proteins for controlling DNA demethylation remains controversial [125–127]. The potential role of the GADD45 proteins in regulating temporal DNA demethylation patterns is intriguing given that *GADD45A* and *GADD45G* display enriched expression specifically in early and late neurogenic cells, respectively [14,62]—temporal windows in which cone versus rod fates are specified. Alternatively, LIN28A, an RNA-binding protein involved in the control of RPC proliferation and neurogliogenesis [128] recruits TET1 to DNA to promote DNA demethylation [129,130]. Furthermore, the CXXC proteins interact with the TET enzymes to maintain and stabilize the demethylated state of specific DNA loci [131]. CXXC4/CXXC5 are both expressed within the developing retina; however, the function of these proteins within the retina remains unknown. Further studies will focus on deepening our understanding of the molecular mechanisms that DNA methylation marks are recognized and targeted for site-specific demethylation in control of retinal development.

## Materials and methods

### Ethics Statement

All experimental procedures were preapproved by the Institutional Animal Care and Use Committee (IACUC) as part of the Division of Comparative Medicine at Washington University in St. Louis School of Medicine under protocol numbers 22-0123 and 24-0374.

### Mice

Postnatal day (P) P0, P21, and 6 weeks old Cre-negative, heterozygous, and Tet conditional knockouts (cKO) were generated using the Tg(Chx10-EGFP/cre,-ALPP)2Clc/J(*Chx10*::Cre-GFP^63^; Tet1^*loxp/loxp*^; Tet2*l*^*oxp/loxp*^; Tet3^*loxp/loxp*^ [42]). To reduce mosaicism of the Cre transgene, Cre+ animals were in-crossed to ensure the presence of two functional copies of the Cre transgene in all breeders and offspring for both tHet and tcKO animals. Control mice (Cre−) were obtained from age-matched animals using Cre− breeders. All mice were housed in a climate-controlled pathogen-free facility on a 14/10 hour light/dark cycle.

### Tissue processing for H&E staining

Eyes were enucleated from euthanized animals and placed in 4% paraformaldehyde (PFA) for 1–2 min before generating a corneal tag by removing a portion of the ventral cornea to enable dorsal-ventral orientation following sectioning. Eyes are then fixed overnight in 4% PFA overnight followed by washing in phosphate-buffered saline (PBS) for 3 min. Eyes are then placed in 70% ethanol at 4 °C until paraffin embedding.

Embedding and sectioning of paraffin blocks and H&E staining was performed following standard protocols by the histology core in the Department of Ophthalmology and Visual Sciences at Washington University.

### Electroretinograms (ERGs)

ERGs were performed as previously described [115]. Briefly, tests were performed on a visual electrodiagnostic system (UTASE3000 with EM for Windows; LKC Technologies, Gaithersburg, MD, USA) while mouse body temperature was maintained at 37 °C ± 0.5 °C with a heating pad controlled by a rectal temperature probe (FHC, Bowdoin, ME, USA). Pupils were dilated with 1.0% atropine sulfate (Bausch & Lomb, Tampa, FL, USA), and dilation and corneal hydration were maintained during testing by positioning the platinum wire loop recording electrodes in a mixture of atropine and 1.25% hydroxypropyl methylcellulose (GONAK; Akorn, Buffalo Grove, IL, USA). Mice were tested without knowledge of genotype. Bilateral flash ERG responses were obtained; the set of recordings displaying larger peak amplitudes were correlated with genotype information for statistical analyses. Differences in peak amplitude response at all light intensities were determined using a Two-way ANOVA and with Geisser–Greenhouse correction, followed by a Dunnett’s multiple comparison tests were performed using GraphPad Prism v10.2.3 (GraphPad Software, La Jolla, CA, USA).

### Immunohistochemistry

Eyes were enucleated from animals and placed in cold 4% PFA for 1 h followed by washing in 1× PBS. Retinas were then dissected to remove the choroid/sclera, RPE, and anterior segment and placed into 30% sucrose in PBS overnight at 4 °C. Retinas were placed into 30% sucrose in PBS:OCT (1:1) overnight at 4 °C and subsequently mounted in Tissue-Tek OCT media (VWR) for sectioning.

Immunohistochemistry was performed following standard protocols. Briefly, slides are air dried and then washed in 1× PBS and placed into blocking solution [(1× PBS, 5% horse serum, 0.2% triton, 0.02% sodium azide, 0.1% bovine serum albumin (BSA)] for 2 h. Slides are placed in the primary antibody diluted in blocking solution overnight at 4 °C (S1 Table). Slides are then washed in 1× PBS plus 0.05% triton three times for 5 min each. Primary antibodies are detected through incubation using fluorescently tagged secondary antibodies diluted 1:500 in blocking buffer for 2 h in the dark. Slides are then washed in 1× PBS plus 0.05% triton and then nuclei are counterstained using DAPI (1:3000 in 1× PBS plus 0.05% triton). Slides are then coverslipped using Vectashield HardSet Antifade Mounting Medium (Vector Labs).

### TUNEL staining

TUNEL staining was performed according to the manufacturer’s protocol (In Situ Cell Death Detection Kit, TMR red, Cat. 12156792910). Briefly, slides were incubated in fixation solution (4% PFA in PBS) for 20 min at room temperature followed by a 30 min wash in PBS. and blocking solution [1× PBS, 5% horse serum, 0.2% triton, 0.02% sodium azide, 0.1% BSA] for 2 min on ice. After two washes with 1× PBS, sections were incubated in the TUNEL reaction mix, containing the Labeling Solution (TMR red labeled nucleotides) and the enzyme Solution (terminal deoxynucleotidyl transferase) for 60 min at 37 °C in dark conditions. Slides were washed three times in 1× PBS. Once dried, slides were coverslipped using Vectashield HardSet Antifade Mounting Medium (Vector Labs).

### EdU experiments

Newborn (P0) mice were injected with EdU (10 µM final concentration) and retinas were harvested at P1 or P14. Retinas are then processed for immunohistochemistry. EdU staining was performed using the Click-IT EdU Alexa Fluor 647 imaging kit (Invitrogen) following manufacturer’s instructions, with slides placed into blocking steps for the immunohistochemistry protocol directly after EdU detection. Nuclei were counterstained with DAPI (1:3000) and coverslipped using Vectashield (Vector Labs).

### Cell counts, fluorescence intensity measures, and statistics

For measuring the retinal thickness, measurements were taken from 1,500 µm both dorsally and ventrally from the optic nerve head (ONH). For cell counts, three images per retina were used to generate an average for each individual replicate. Replicate samples are from independent animals. Cell counts of control and Tet mutant retinas were made from a 200 × 200 µm area. Cell proportions were calculated by determining the number of marker-positive cells divided by the total DAPI+ nuclei within the region of interest. For nuclear architecture and chromatin distribution assessments, chromocenters of 100 cells were manually counted in each section. Cell proportions in EdU experiments were calculated by determining the number of double-positive cells (marker+/EdU+) divided by the total number of EdU+ cells in the whole image. Measures of retinal layers thickness, photoreceptor layers, fluorescence intensity measures, and cell counts were performed using Fiji software 1.54f [132]. Statistical analyses were performed with GraphPad Prism v10.2.3 (GraphPad Software, La Jolla, CA, USA).

### Imaging

Immunohistological data from H&E staining was imaged and photographed on the Zeiss Axio Observer inverted microscope coupled with an Axiocam 208 color camera (Zeiss). Immunohistochemical data from fluorescence labeling was imaged and photographed using an LSM800 confocal (Zeiss). 63× microphotographs were taken using the Airyscan imaging tool. Figure preparation was performed using Adobe Illustrator (Adobe, San Jose, CA). Contrast and brightness were minimally adjusted using Fiji software 1.54f [132].

### Mouse Retina RNA and DNA isolation

RNA and DNA were isolated following a modified protocol from TRIZOL Reagent isolation protocol. Briefly, retinal samples were placed in 1 mL TRIZOL solution and homogenized. 200 µL of chloroform was added to homogenized TRIZOL solution, mixed by vortexing, and incubated for 3 min at RT. Lysates were centrifuged for 10 min at 4 °C at max speed (16,000*g*). The aqueous phase with RNA was transferred into a fresh tube without disturbing the interphase. The rest of the volume, containing DNA and proteins was washed with 300 µL 100% ethanol, inverted several times, and then centrifuged for 5 min at 4 °C at 2,000*g*. The supernatant containing proteins was carefully removed to not disturb the precipitated DNA pellet.

### Mouse Retina RNA extraction

RNA was extracted following standard protocol of the RNA Clean & Concentrator-5 (Zymo Research). Briefly, an equal volume of 100% ethanol was added to the aqueous phase containing RNA from the previous step. Samples were transferred to Zymo-Spin IC Column in a collection tube and centrifuged at 16,000*g*. After discarding the flow-through, an in-column DNAse I treatment was performed. 400 µL of RNA Prep buffer was added to the column and centrifuged at 16,000*g*. After discarding the flow-through, two subsequent washes with RNA wash buffer were made. Then, the column is transferred into an RNAse-free tube and RNA eluted from the column using 30 µL DNase/RNase-free water. RNA concentrations were measured using a Qubit fluorometer.

### Mouse Retina DNA extraction

After discarding the supernatant containing proteins, the DNA pellet was washed in 1 mL of 100 mM sodium citrate in 10% ethanol and incubated for 30 min at RT followed by centrifugation 5 min at 4 °C at 2,000*g* and removal of the resulting supernatant. This step is repeated twice. 1.5 mL of 75% ethanol was added to the DNA pellet and incubated for 20 min at RT using occasional mixing during the 20-min incubation. The supernatant is discarded, and the DNA pellet is air-dried for 10 min. 100 µL of 8 mM NaOH in 1 mM EDTA pH 7–8 was added to resuspend the DNA pellet. After that, samples are centrifuged for 10 min at 4 °C and max speed (16,000*g*). The supernatant is transferred to a new tube and stored at −20 °C. DNA concentrations were measured in a Qubit fluorometer.

### Nuclei Isolation and single-nucleus RNA-sequencing (snRNA-seq) library preparation

Dissected retina tissue was dissociated on ice in 500 µl cold Homogenization Buffer (25 mM KCl, 5 mM MgCl_2_, 10 mM Tris-HCl, 1 mM DTT, complete Mini Protease Inhibitor Cocktail (Roche)) with RNase inhibitors (200 U/mL RNasin (Promega), 200 U/mL SUPERaseIn (Invitrogen)) using a Dounce homogenizer, with 15 strokes each of pestles A and B. The lysate was strained through a 40 µm cell strainer, which was then rinsed with 9 mL additional Homogenization Buffer. The strained lysate was centrifuged for 5 min at 500*g* in a 4 °C swinging-bucket centrifuge. After discarding the supernatant, the nuclei pellet was gently resuspended in 1 mL cold Resuspension Buffer (25 mM KCl, 3 mM MgCl_2_, 50 mM Tris-HCl, 1 mM DTT) with RNase inhibitors (200 U/mL RNasin (Promega), 200 U/mL SUPERaseIn (Invitrogen)) using a wide-bore pipet tip. Nuclear concentration was quantified by Countess. Appropriate numbers of nuclei were then processed according to the PIPseq T20 3′ Single Cell RNA Kit v4.0 (Fluent BioSciences). Completed libraries were sequenced on a NovaSeq X Plus (Illumina) and reads processed using pipseeker (v3.3.0) and aligned to the GRCm39 reference genome.

Libraries were sequenced on the Illumina NovaSeq X Plus using 2 × 150 bp paired-end reads.

### bACE-seq library preparation

bACE-Seq libraries were made using the protocol from [96] with minor modifications. In brief, 10–15 ng of RNAse-treated, TRIZOL-purified gDNA was used for each sample. Samples were diluted to 10 µL in MilliQ water and pre-heated by incubating at 50 °C for 20 min on a thermocycler with a heated lid set to 95 °C. 32.5 µL of CT Conversion Reagent (Zymo EZ DNA Methylation Direct Kit) was then added to each sample before running the following program on a thermocycler (98 °C for 8 min, 64 °C for 105 min, followed by 98 °C for 8 min, 64 °C for 105 min), with the lid set to 95 °C. Following the bisulfite reaction, the tubes were placed at −20 °C overnight, or for 16 h. The bisulfite converted DNA was then purified using the Zymo EZ Methylation Direct Kit, eluting with 17 µL of MilliQ water for a final elution volume of 16 µL.

Each sample was incubated at 95 °C for 3 min on a thermocycler, with the lid set to 105 °C. The samples were then immediately placed into a dry-ice ethanol bath to snap cool them and were left in the cooling bath for 5 min. The samples were then placed on a pre-chilled PCR rack and kept on ice. 8 µL of premixed APOBEC reaction mixture (2.4 µL APOBEC reaction buffer (E7134A, NEB), 0.48 µL APOBEC (E7133AA, NEB), 0.48 µL BSA (B9000S, NEB), water to 8 µL) was added to each tube and samples were incubated at 37 °C on a thermocycler with the lid set to 95 °C. After 30 min, the samples were mixed and placed back on the thermocycler for two and a half hours at 37 °C with the lid set to 95 °C. DNA was purified from the reaction using 2× Agencourt AMPure XP SPRI beads (add 2× volume of SPRI beads, incubate at room temperature for 10 min, place samples on magnet and wash two times with fresh 80% ethanol, dry for 3 min, and eluted in 9 µL Low EDTA TE (IDT).

Sequencing libraries were constructed using the xGen Adaptase Module with minor modification. 250 nM of random stubby index primer was added to each sample (xGen Adaptase Module, P5L_AD002_H). Samples were incubated at 95 °C on a thermocycler with the lid set to 105 °C for 3 min, then cooled on ice for 2 min. 10 µL of BST-based random priming mixture (2 µL 10× ISO AMP Buffer II (B0374S, NEB), 0.8 µL BST 3.0 (M0374S, NEB), 1.2 µL 200 mM MgCl_2_, 2.8 µL 10 mM dNTP, 3.2 µL water) was then added to each sample and incubated at 60 °C for 1 h on a thermocycler with the lid set to 95 °C. Fragment ends were blunted and dephosphorylated by adding 2 µL Exonuclease I (M0293L, NEB) and 1 µL shrimp alkaline phosphatase (M0371S, NEB) to each sample. The samples were then incubated at 37 °C for 30 min and purified using a 1.6× SPRI beads (add 1.6× volume of SPRI beads, incubate at room temperature for 10 min, place samples on magnet and wash two times with fresh 80% ethanol, dry for 3 min, elute) and eluted in 10 µL Low EDTA TE (IDT). In brief, samples were denatured by incubating at 95 °C on a thermocycler with the lid set to 105 °C for 3 min, then cooled on ice for 2 min. Samples were indexed and amplified using the PCR protocol in the xGen Adaptase Module using 21 cycles. The indexed samples were purified using 1.6× SPRI beads (add 1.6× volume of SPRI beads, incubate at room temperature for 10 min, place samples on magnet and wash two times with fresh 80% ethanol, dry for 3 min, elute) and eluted in 12 µL Low EDTA TE (IDT).

Libraries were initially sequenced by spike-in low-pass sequencing 2 × 150 bp on an Illumina MiniSeq (~100k reads per sample) to ensure that libraries were high quality (reasonable mapping and APOBEC conversion efficiencies) before being sequenced using 2 × 150 paired-end reads on an Illumina NovaSeq X Plus.

### WGBS library preparation

Genomic DNA was quantified using the Qubit fluorometer. 200 ng of gDNA, including 0.2% Lambda DNA (N6-methyladenine-free; NEB) was fragmented in a final volume of 50 µL using the Covaris LE220 targeting ~350 bp inserts. A 1.5× AMPure clean-up was utilized after fragmentation to concentrate the sample. The fragmented gDNA was bisulfite converted with the EZ-96 DNA Methylation-Gold Mag Prep Kit (Zymo Research, Cat # D5043) according to the manufacturer’s recommendations. Bisulfite converted DNA was quantitated on the Qubit Fluorometer using the ssDNA Assay Kit (Thermo Fisher Scientific, Cat #Q10212). Whole genome bisulfite libraries were constructed with ~100 ng of converted DNA using the xGen Methyl-Seq Library Prep Kit (Integrated DNA Technologies, Cat # 10009825) using unique dual indexes (IDT, 0008053) and eight PCR cycles for incorporation and amplification of indexed libraries, followed by a final 0.85× AMPure cleanup. Final libraries are quality checked for average library size and concentration. Molar concentration of libraries was determined using the KAPA Library Quantification kit (Roche Diagnostics). Libraries were sequenced on NovaSeq X using 150 bp paired-end reads.

### RNA-seq library preparation

For RNA-seq library preparation, SMARTer Stranded Total RNA High Input (RiboGone Mammalian; Takara) was used. Briefly, extracted RNA first undergoes ribosomal RNA removal. Then, we proceeded with cDNA synthesis and purification. Following, RNA-seq library was amplified by PCR. Total RNA was captured and purified from RNA samples using Illumina TruSeq stranded primers. RNA-libraries concentrations were measured in a Qubit fluorometer and using the Agilent 2100 Bioanalyzer. 9 libraries were pooled and sequenced using the NovaSeq X Plus 300 cycles system with ~300 million paired-reads per run, resulting in between 40 million and 55 million reads per library.

### Sequencing Analysis

*bACE-seq*—Data were processed similarly to [96] with minor modification. Paired-end sequencing reads were processed with TrimGalore (v0.4.4_dev; https://github.com/FelixKrueger/TrimGalore) to remove adapters and low-quality sequences using the parameters --paired --clip_R1 16 --clip_R2 16. The trimmed reads were independently aligned to the mouse genome (mm10) using Bismark (v0.18.2). Read 1 (R1) was aligned with Bowtie2 in PBAT mode (--pbat flag) and the following settings: -q --score-min L,0,-0.2 -p 4 --reorder --ignore-quals --no-mixed --no-discordant --dovetail --maxins 500 --directional. Read 2 (R2) was mapped using Bowtie2 with default parameters. Aligned reads were filtered to retain only those with a mapping quality score ≥ 10 using Samtools (v1.15.1) and PCR duplicates were removed using Picard’s MarkDuplicates tool (GATK v4.1.3). CpG methylation levels were extracted with MethylDackel (v0.6.1; https://github.com/dpryan79/MethylDackel) using the --CHG and --CHH flags alongside default settings. Finally, strand-specific read counts were summed for both the converted and unconverted reads across strands. Percentage of methylation was then calculated for converted read counts divided by the total read counts for each CpG site.

*WGBS*—WGBS data were processed using the same pipeline as described for bACE-seq analysis (above), with modifications to the alignment steps. Reads were aligned to the mouse genome (mm10) using Bismark (v0.18.2) [133] with default alignment parameters (PBAT mode turned off).

*Differentially Methylated Region (DMR) Analysis*—DMRs were identified using DSS (v2.48.0) [134]. The DMLtest function was applied with smoothing=TRUE and default parameters. DMRs were called with a p-value threshold of 0.01. To define DMRs, a minimum length of 50 bp and at least 3 CpG sites were required, with DMRs within 50 bp merged (following DSS default settings). DMR annotation was performed using ChIPseeker (v1.36.0) [135], with the TSS Region set to ± 2,000 bp and default settings. Mouse gene annotations from TxDb.Mmusculus.UCSC.mm10.knownGene were used for the annotation process.

**bACE-seq and WGBS-seq Data Integration*—*For each CpG site, 5mC and 5hmC levels were estimated using MLML, which calculates maximum likelihood estimates by integrating data from bACE-seq (5hmC) and WGBS-seq (5mC + 5hmC). MLML [99] was applied with a significance threshold of *α* = 0.05 for the binomial test at each CpG site, and similarly for CpH sites in non-CpG analyses.

#### RNAseq.

Quality control of raw sequencing reads was performed using FastQC (v0.11.9) (https://www.bioinformatics.babraham.ac.uk/projects/fastqc/?utm_source), with summaries generated by MultiQC (v1.19) [136]. Adapter trimming and removal of low-quality bases were conducted using Trim Galore (v0.6.7) (https://www.bioinformatics.babraham.ac.uk/projects/trim_galore/). Trimmed reads were aligned to the mm10 reference genomes, using STAR (v2.7.0f; with default parameters optimized for paired-end data, followed by post-alignment processing, including sorting and indexing of BAM files, using Samtools (v1.9) [137. Gene-level quantification was performed using HTSeq (v0.11.2) [138], and genome-wide read coverage was visualized using deepTools (v3.5) [139]. Differential expression analysis was conducted in edgeR (v4.2.2) [140] within R (v4.4.1), employing TMM normalization and statistical modeling, with significance thresholds set at |log2FC| ≥ 1 and FDR < 0.01. Heatmaps were generated using pheatmap (v1.0.12) https://CRAN.R-project.org/package=pheatmap), and GO enrichment analysis was performed with clusterProfiler (v4.12.6) [141].

#### snRNA-seq analysis.

To lessen the effects of ambient RNA contamination, resulting matrices from pipseeker outputs were processed using Cellbender v.0.3.0 [142]. The priors used for the --expected-cells and --total-droplets-included parameters were 1,000 and 350,000, respectively. A “full” model was used for both samples with the model dimensions set to --z-dim 256 and --z-layers 2048. Each model was trained to 150 epochs using a learning rate of 1 × 10^−7. Only cells with a 0.99 probability were used in the downstream analysis.

Resulting matrices from Cellbender were imported into Monocle3 [143,144] for single-cell analysis. Cells containing greater than 500 and less than 10,000 transcripts with less than 20% mitochondrial transcripts were utilized for downstream analyses. Dimension reduction was performed using a preprocessed matrix with PCA followed by UMAP dimension reduction using the first 16 PCA dimensions. Cell type annotations were performed using marker gene expression within clusters for known marker genes for retinal cell types as determined previously [14]. Differential expression analysis by genotype was performed using the Monocle3 fit_models function performed on all genes expressed within at least 100 cells and a q-value threshold of 1e−20.

## Supporting information

S1 Fig Characterization of the morphological changes driven by TET enzyme loss-of-function. Related to Fig 1. **(A)** Whole retina, outer nuclear layer (ONL) and inner nuclear layer (INL) thickness measurements at different eccentricities from the optic nerve head (ONH) in P21 retinas. Results display the mean + SEM for *n* = 3 for each genotype. **(B)** H&E staining of an allelic series of TET conditional mutant retinas at 6-weeks of age. **(C)** Whole retina, outer nuclear layer (ONL) and inner nuclear layer (INL) thickness measurements at different eccentricities from the optic nerve head (ONH) in 6-week-old retinas. Results display the mean + SEM for *n* = 3 for each genotype. Scale bars: 100 µm. Data files for graphs available in S2 Data.
(TIF)

S2 Fig Immunohistochemical characterizations of cell types across the TET enzyme allelic series of conditional mutants. Related to Fig 2. **(A)** Immunohistochemistry for retinal ganglion cells (RBPMS), amacrine cells (PAX6); horizontal cells (CALB1); bipolar cells (VSX2); Müller glia cells (LHX2) markers that show changes in cell proportions of some retinal cell types when TET enzymes are absent. **(B)** Immunohistochemistry for amacrine cells (TFAP2A). **(C)** Graph showing cell counts of TFAP2A+ cell proportions across genotypes. **(D)** Immunohistochemistry for bipolar cells (OTX2). **(E)** Graph showing cell counts of OTX2+ cell proportions across genotypes. Results display the mean + SEM for *n* = 3 for each genotype. Statistics are the result of Unpaired t-tests. * *p* < 0.05. Scale bars: 100 µm. Data files for graphs available in S5 Data.
(TIF)

S3 Fig Loss of TET enzymes results in high levels of RPC transcription factors co-expression within presumptive Müller glia. Related to Fig 2I and 2J. **(A–D)** Immunohistochemistry for bipolar cells (VSX2); amacrine cells (PAX6) and Müller glia cells (LHX2) markers in (A, C) Cre− and (B, D) Tet tcKO retinas indicating prominent co-localization of VSX2, PAX6, and LHX2 and in presumptive Müller glia in Tet tcKO retinas. **(E)** Graph showing the number of VSX2, PAX6 double positive cells across control and Tet tcKO retinas. Results display the mean + SEM for *n* = 3 for each genotype. Statistics are the result of an Unpaired *t* test. * *p* < 0.05. Scale bars: 100 µm. Data files for graphs available in S6 Data.
(TIF)

S4 Fig Changes in cell proportions, distribution, glial cell morphology, and cell death in TET enzyme mutant retinas. Related to Fig 2. **(A)** Immunohistochemistry for Müller glia cells (GS and GFAP) in P21 and 6 weeks old retinas. **(B)** Immunohistochemistry for microglial cells (IBA1) show changes in cell proportions of microglial cells when TET enzymes are absent. **(C)** Results display the mean + SEM for *n* = 5 for each genotype. Statistics are the result of an Ordinary One-Way ANOVA, followed by a Dunnett’s multiple comparisons test. **** *p* < 0.0001. **(D)** Immunohistochemistry for microglial cells (IBA1) showing the comparisons between tHet and Tet1/2/3 cKO retinas and the layers that were considered for cell counts in E and F. **(E, F)** Graphs showing the difference in microglial cell localization in tHet and Tet tcKO retinas. Results display the mean + SEM for *n* = 5 for each genotype comparing (E) combined plexiform or nuclear layers, and (F) microglia localization within individual nuclear layers (ONL, INL, and GCL). Statistics are the result of an Unpaired *t* test or Mann–Whitney test. (E) ** *p* < 0.01. (F) * *p* < 0.05; ** *p* < 0.01. **(G)** Immunohistochemistry for microglia cells (IBA1) and apoptotic cells (TUNEL) in P21 Cre− and Tet tcKO retinas. **(H)** Results display the mean + SEM for *n* = 3 for each genotype. Statistics are the result of an Unpaired *t* test. * *p* < 0.05. Scale bars: 100 µm. Data files for graphs available in S7 Data.
(TIF)

S5 Fig Altered photoreceptor fate specification in TET mutant retinas. Related to Fig 3. **(A)** Immunohistochemistry for cone-photoreceptor (CRX, RXRγ, and ARR3) and rod-photoreceptor (CRX, NRL, and NR2E3) markers indicate alterations in photoreceptor cells proportions when TET enzymes are absent. **(B)** Graphs showing the significant decrease in the number of photoreceptor cell nuclear layers in the ONL comparing Cre−, tHet, and Tet tcKO H&E-stained retinas both at P21 and 6 weeks. Results display the mean + SEM for *n* = 3 for each genotype. Statistics are the result of One-way ANOVA followed by a Tukey’s comparisons test * *p* < 0.05; ** *p* < 0.01. **(C)** Graphs showing the contribution of rod-photoreceptors and cone-photoreceptors to the total number of photoreceptor cells across genotypes. **(D)** Changes in the location of cone-photoreceptor nuclei in the ONL across genotypes, indicating presence of cone nuclei below the midline of the ONL. Scale bars: 100 µm. Data files for graphs available in S3 Data.
(TIF)

S6 Fig Tet tcKO results in altered retinal synapse structures. Related to Fig 1E. **(A, B)** Immunohistochemistry for synaptic markers BASSOON and CALRETININ that show the disruption of the OPL and IPL respectively, at P21 and 6 weeks. **(C)** Graph showing the number of ribbon synapses in the OPL labeled by BASSOON. Results display the mean + SEM for *n* = 3 for each genotype. Statistics are the result of a One-Way ANOVA, followed by a Tukey’s comparisons test; ** *p* < 0.01; *** *p* < 0.001. **(D)** Immunohistochemistry for cone-photoreceptors (ARR3) and the synaptic marker BASSOON in P21 retinas showing the loss of cone ribbon synapses in Tet tcKO retinas. Scale bars: (A, B) 100 µm; (D) 10 µm. Data files for graphs available in S9 Data.
(TIF)

S7 Fig Tet tcKO alters photoreceptor nuclear architecture/chromatin condensation. **(A)** Immunohistochemistry for nuclei (DAPI) and cone-photoreceptors (ARR3) in P21 retinas. **(B)** Graph showing the mean number of chromocenters/nuclei across genotypes. Results display the mean + SEM for *n* = 3 for each genotype. Statistics are the result of an Unpaired *t* test. ** *p* < 0.01. **(C)** Graphs showing the changes in the number of chromocenters/nuclei per genotype. Statistics are the result of a Mann–Whitney test. **** *p* < 0.0001. **(D)** Graphs showing the differences in proportion (%) of nuclei containing 1–6 chromocenters across genotypes. Scale bar: 20 µm. Data files for graphs available in S10 Data.
(TIF)

S8 Fig Tet tcKO results in alterations to the total number of progenitor cells and photoreceptors. Related to Fig 4. **(A)** Graph showing the total number of VSX2+ and EdU+ cells after P0-P1 EdU pulse. **(B, C)** Graphs showing the proportion (PH3+/EdU+) and total number of PH3+ cells P0–P1 EdU pulse. **(D)** Immunohistochemistry showing the labeling of mitotic cells (PH3+ cells). **(E, G)** Graphs showing the total number of CRX+ cells after (E) P0-P1 and (G) P0-P14 EdU pulses. **(F, H)** Graphs showing the total number of NR2E3+ and EdU+ cells after (F) P0–P1 and (H) P0–P14 EdU pulses. **(I)** Graph showing the total number of RXRγ+ cells after P0–P14 EdU pulse. Results display the mean + SEM for *n* = 5 (P0-P14) or *n* = 6 (P0-P1) for each genotype. Statistics are the result of two-tailed Unpaired *t* test; ns: nonsignificant; * *p* < 0.05, *** *p* < 0.001; **** *p* < 0.0001. Scale bars: 100 µm. Data files for graphs available in S12 Data.
(TIF)

S9 Fig Related to Fig 5. Transcriptional profiling of Tet tcKO mutant retinas. **(A)** Read depth and quality score metrics for bulk RNAseq samples. **(B)** Total transcript reads for Tet1 (left), Tet2 (middle) and Tet3 (right) across genotypes. **(C)** Genome browser tracks indicating read alignment to floxed exons in Cre− controls (green) and Tet tcKO replicates (purple) for Tet1 (left), Tet2 (middle), and Tet3 (right). **(D)** Total counts per million (CPM) for reads aligning to floxed exons across genotypes. **(E)** Splicing efficiency of floxed exons for Tet1 (left), Tet2 (middle), and Tet3 (right). Splicing efficiency is determined by the ratio of the number of reads that splice into the floxed exon divided by the sum of the splice reads including and excluding the floxed exon. **(F)** Heatmap of all differentially expressed transcripts (Fold Change > 2; *q*-value < 0.01) between Cre− and tHet RNAseq pairwise comparisons across all RNA-seq replicates. **(G–I)** UMAP dimension reductions of the full snRNAseq datasets with cells colored by (G) genotype, (H) annotated cell type, and (I) total transcripts detected per cell. Data files for graphs available in S13 Data.
(TIF)

S10 Fig Related to Fig 5. Gene modules of differentially expressed genes highlight changes in photoreceptor gene expression patterns. **(A)** UMAP dimension reduction of the mouse retinal development scRNAseq dataset from [14] with cells colored by annotated cell type. **(B, C)** Heatmaps displaying relative z-scores of differentially expressed transcript gene modules from (B) P21 Tet tcKO RNAseq and (C) P21 Tet tcKO snRNAseq across annotated cell types in the mouse retinal development scRNAseq dataset. **(D, E)** UMAP dimension reductions of the [14] dataset with cells colored by relative z-scores of differentially expressed transcripts as gene modules from (D) P21 Tet tcKO RNAseq and (E) P21 Tet tcKO snRNAseq datasets. **(F)** Heatmaps of cone photoreceptors transcripts that display developmentally regulated expression enrichment across (top) developmental age and (bottom) retinal cell types from [14]. **(G)** Boxplots of the log2 fold-change of designated developmental (Early) or mature cone transcripts in Tet tcKO bulk RNA-seq and snRNA-seq datasets compared to Cre− controls, highlighting the increased expression of developmental cone transcript expression in Tet tcKO retinas. Data files for graphs available in S15 Data.
(TIF)

S11 Fig Methylation profiling in control and Tet tcKO retinas. Related to Fig 6. **(A)** Graphic displaying results of WGBS and bACE-seq to distinguish 5mC and 5hmC marks. **(B)** Comparisons of the temporal WGBS methylation patterns across retinal development within the ±5kb of the TSS for up- and down-regulated transcripts from Tet tcKO RNAseq experiments. **(C)** Graph highlighting estimated APOBEC3A failed conversion rates utilizing *pCal7* plasmid spiked-in to each bACE-seq reaction. Statistics represent results of a student *t* test (ns, not significant). **(D, E)** Summary statistics for WGBS and bACE-seq experiments indicating total reads and average coverage for each sample. **(F)** MLML Output statistics for distinguishing 5hmC and 5mC marks. **(G, H)** Scatterplots assessing the correlation RNA expression levels and average 5hmC levels across the (G) gene body or (H) promoter regions for all genes. Individual genes are colored by differential expression in P21 RNA-seq comparisons between P21 Cre− and Tet tcKO retinas. **(I, J)** Line plots showing correlation coefficients of P21 Tet tcKO or Cre− control WGBS with temporal WGBS methylation profiles across (I) accessible DNA and (J) promoters for ATAC peaks that display a >10% decrease in methylation levels across development. **(K, L)** Boxplots of average methylation profiles for differentially hypomethylated regions identified in comparisons between (K) Cones vs. Rods and (L) Cones vs. *rd7* Rods in P21 Cre− and TET tckO retinal samples and sorted cones, rods, and *rd7* rods. **(M)** Genomic feature distribution for DMRs from WGBS and bACEseq analyses. **(N)** Boxplots of the change in 5hmC levels (Cre− minus Tet tcKO) between P21 Cre− and Tet tcKO retinal samples, highlighting the significant loss of enhancer methylation in Tet tcKO retinas. Data files for graphs available in S16 and S18 Data — https://doi.org/10.6084/m9.figshare.29575223.v1.
(TIF)

S12 Fig Overlap of 5mC, 5hmC, and snATAC peaks within photoreceptor gene loci. Related to Fig 6. IGV genome tracks of photoreceptor transcription factor (left), rod photoreceptor (middle) and cone photoreceptor (right) gene loci. DMRs are shown for 5mC (maroon) and 5hmC (green) with the height of the bar representing the average difference in methylation between Tet tcKO and Cre− WGBS and bACE-seq, respectively. 5mC and 5hmC tracks indicate direction of DMR, with bars above or below the gray equivalence lines in each track indicating gain or loss of methylation, respectively. snATAC peak track (bottom, blue) indicates called peaks from the retinal development snATAC-seq studies in [102].
(TIF)

S1 Table List of primary antibodies used throughout the study, including concentration for which the antibodies were utilized. (XLSX)

S2 Table RNA-seq results indicating normalized transcript expression level across replicate samples, and results of pairwise differential expression tests. (CSV)

S3 Table Table of GO-pathway analyses for Biological Processes of differentially expressed transcripts in Tet tcKO P21 retinas. GO results are provided for up- and down-regulated transcript analyses as separate sheets.
(XLSX)

S4 Table Differential transcript analysis of P21 Tet tcKO and Cre− snRNA-seq results indicating directio n and fold-change of transcript differential expression. (CSV)

S5 Table Table indicating the differentially methylated regions (DMRs) in WGBS analyses. Chromosome coordinates, mean methylation levels of CpGs across genotypes, and percentage of methylation differences are all provided.
(XLSX)

S6 Table Table indicating the differentially methylated regions (DMRs) in bACE-seq analyses. Chromosome coordinates, mean methylation levels of CpGs across genotypes, and percentage of methylation differences are all provided.
(XLSX)

S1 Data Data file for Fig 1C, 1E–1K, providing results of bACE-seq, retinal thickness measurements, and ERG experiments. (XLSX)

S2 Data Data file for Fig S1A and S1C retinal thickness measurements across retinas. (XLSX)

S3 Data Data file for Fig S5B indicating average number of photoreceptor layers observed across genotypes and ages. Values represent the mean number of photoreceptor layers across three individual images for each replicate. (XLSX)

S4 Data Data file for Fig 2B, 2D, 2F, 2H and 2J indicating the percentage of cells co-staining for indicated markers or total number of marker positive cells. Values represent the mean of three images from individual retinas.  (XLSX)

S5 Data Data file for Fig S2C and S2E detailing the percentage of cells co-staining with the indicated marker. Values represent the mean of three images for each replicate sample. (XLSX)

S6 Data Data file for Fig S3E highlighting the total number of PAX6+/VSX2+ cells across replicates. Values indicate the mean number of cells across three representative images for each replicate. (XLSX)

S7 Data Data file for Fig S4C, S4E, S4F, and S4H detailing the mean number of IBA1+ cells across three images per replicate (S4C, S4E, S4F) or the mean TUNEL fluorescence across three separate images per retina (S4H). (XLSX)

S8 Data Data file for Fig 3Y indicating the percentage of marker positive cells across replicates. Values represent the average across three individual images for each replicate sample. (XLSX)

S9 Data Data file for Fig S6C highlighting the average number of Bassoon-labeled ribbon synapses across three representative images for each replicate retina. (XLSX)

S10 Data Data file for Fig S7B and S7C detailing the average number of chromocenters per nuclei across 300 photoreceptor nuclei for each replicate sample (S7B) or the number of chromotcenters for each individual cell (S7C). (XLSX)

S11 Data Data file for Fig 4C, 4E, 4G, 4J, 4L, and 4N indicating the percentage of EdU+ cells co-stained with indicated marker. Values represent the mean of three individual images for each replicate. Values for Fig 4C indicate the percentage of EdU+ cells that fail to co-express VSX2.   (XLSX)

S12 Data Data file for Fig S8A–S8C, S8E, S8G–S8I. Values present the average number or average percentage of cells that are positive for indicated marker(s) calculated from three separate images for each replicate. (XLSX)

S13 Data Data file for Fig S9B, S9D, and S9E highlighting either the normalized RNA-sequencing reads that map to floxed exons (S9D) or percentage of reads that splice around the floxed exons (S9E) for each RNA sequencing replicate. (XLSX)

S14 Data Data file for Fig 5H indicating the fold change in bulk RNA-seq experiments of all transcripts that are either Up-regulated, Down-regulated, or unchanged in snRNA-seq experiments. (XLSX)

S15 Data Data file for Fig S10G displaying the log2 Fold Change of early or late cone transcripts in Bulk RNA-seq experiments or in snRNA-seq experiements. (XLSX)

S16 Data Data file for Fig S11C, S11K, and S11L. (S11C) Average conversion failure rates for bACE-seq replicates are provided based on remaining cytosines in sequencing reads of pCal7 vector spiked into genomic DNA samples prior to bACE-seq reactions. (S11K) Average Methylation (5mC + 5hmC) levels across hypomethylated regions in cones compared to rods in WGBS experiments. (S11L) Average Methylation (5mC + 5hmC) across hypomethylated regions in cones compared to rd7 rods in WGBS experiments.  (XLSX)

S17 Data Data file for Fig 6C, 6E, 6J, 6K, 6N–6S. (6C) Percentage of methylated cytosines across di/tri-nucleotide sequences from WGBS datasets. (6E) Percentage of cytosines displaying 5hmC from bACE-seq experiments. (6J, 6K) Fraction of deconvoluted 5mC or 5hmC levels at promoter sequences detected in methylation profiling experiments of control samples for bulk RNA-seq, differentially expressed transcripts. (6N, 6O) Fraction of deconvoluted 5mC or 5hmC levels across gene bodies detected in methylation profiling experiments of control samples for bulk RNA-seq, differentially expressed transcripts. (6P) Gene body 5hmC levels and transcript expression in Cre- samples, including the quartile designation for each transcript based on RNA-seq expression levels. (6Q) Promoter 5hmC levels and transcript expression in Cre- samples, including the quartile designation for each transcript in based on RNA-seq expression levels. (6R) Average Methylation (5mC + 5hmC) levels across hypomethylated regions in rods compared to cones in WGBS experiments. (6S) Average Methylation (5mC + 5hmC) levels across hypomethylated regions in rods compared to rd7 rods in WGBS experiments. (XLSX)

## Abbreviations

**:**
BER: base-excision repair
BSA: bovine serum albumin
CREs: *cis*-regulatory elements
DMR: differentially methylated region
DNMTs: de-novo methyltransferases
ERGs: electroretinograms
FDR: False Discovery Rate
GO: Gene Ontology
GRNs: gene regulatory networks
GS: glutamine synthetase
INL: inner nuclear layer
IPL: inner plexiform layer
ONH: optic nerve head
ONL: outer nuclear layer
OPL: outer plexiform layer
PBS: phosphate-buffered saline
PFA: paraformaldehyde
PIPseq: particle-templated instant partition sequencing
RGCs: retinal ganglion cells
RNA-seq: RNA-sequencing
RPCs: retinal progenitor cells
snATAC: single-nucleus Assay for Transposase Accessible Chromatin
snRNA-seq: single-nucleus RNA-sequencing
TDG: Thymine DNA-Glycosylase
TET: ten-eleven translocation
TSS: transcription start sites
WGBS: Whole Genome Bisulfite Sequencing
5-caC: 5-carboxylcytosine
5-fC: 5-formilcytosine
5hmC: 5-hydroxymethylcytosine
5mC: 5-methylcytosine
