# Supplementary figures and images for: Active DNA demethylation upstream of rod-photoreceptor fate determination is required for retinal development
Source: PLoS Biol. 2025 Aug 4;23(8):e3003332. doi: 10.1371/journal.pbio.3003332 (PMC12407554; doi:10.1371/journal.pbio.3003332)

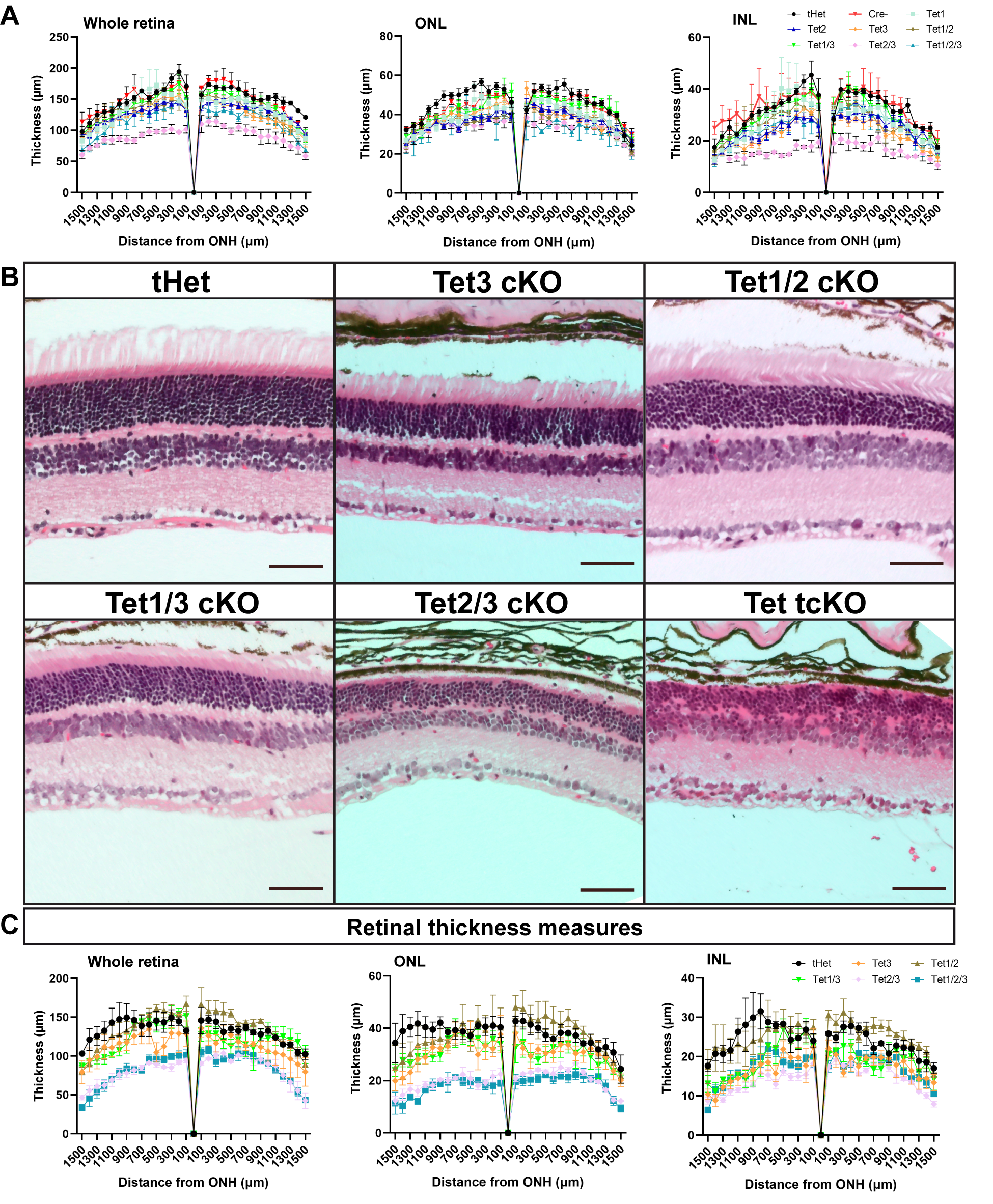

Supplement: S1 Fig — (A) Whole retina, outer nuclear layer (ONL) and inner nuclear layer (INL) thickness measurements at different eccentricities from the optic nerve head (ONH) in P21 retinas. Results display the mean + SEM for n = 3 for each genotype. (B) H&E staining of an allelic series of TET conditional mutant retinas at 6-weeks of age. (C) Whole retina, outer nuclear layer (ONL) and inner nuclear layer (INL) thickness measurements at different eccentricities from the optic nerve head (ONH) in 6-week-old retinas. Results display the mean + SEM for n = 3 for each genotype. Scale bars: 100 µm. Data files for graphs available in S2 Data. (TIF) [file pbio.3003332.s001.tif]

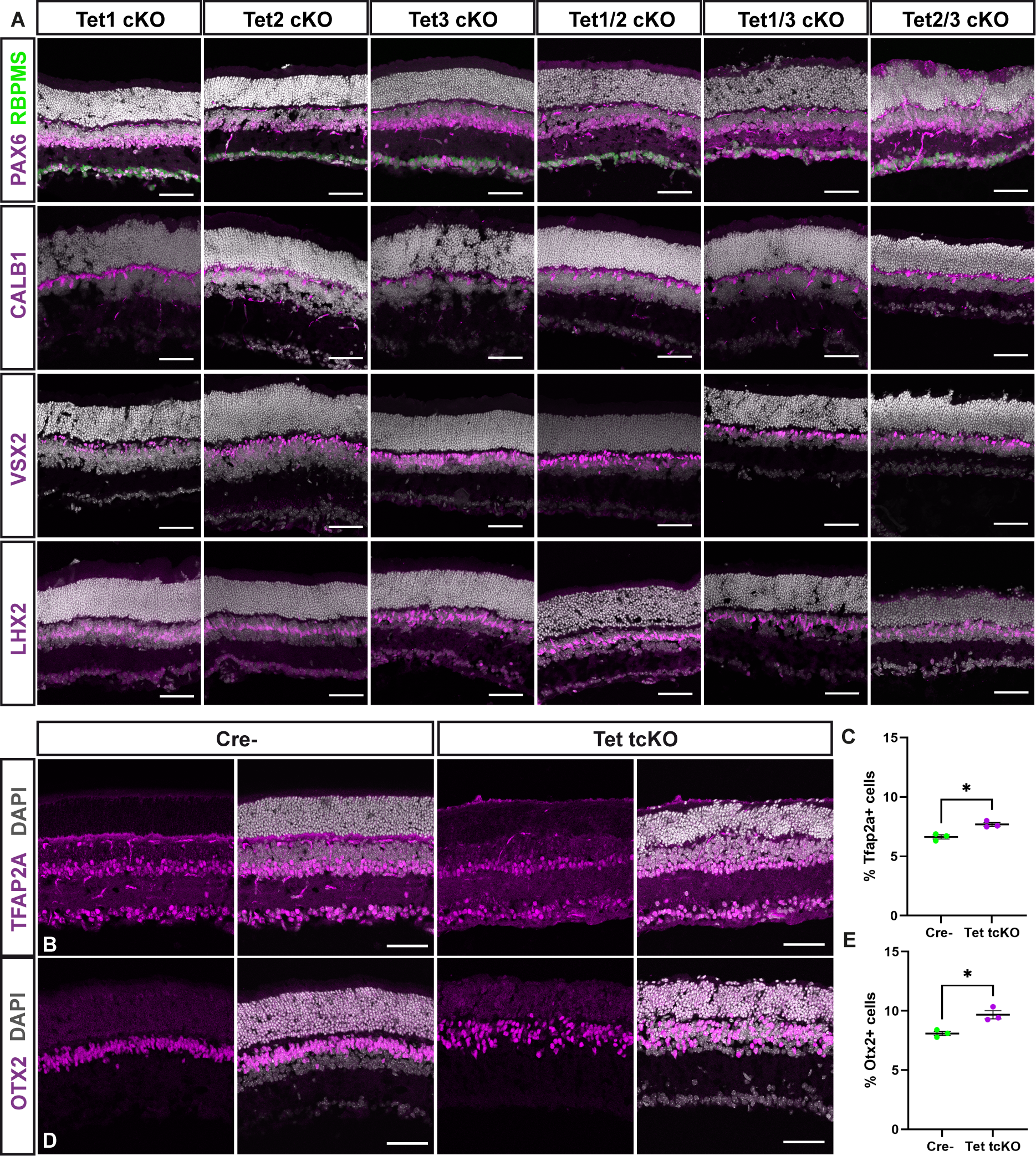

Supplement: S2 Fig — (A) Immunohistochemistry for retinal ganglion cells (RBPMS), amacrine cells (PAX6); horizontal cells (CALB1); bipolar cells (VSX2); Müller glia cells (LHX2) markers that show changes in cell proportions of some retinal cell types when TET enzymes are absent. (B) Immunohistochemistry for amacrine cells (TFAP2A). (C) Graph showing cell counts of TFAP2A+ cell proportions across genotypes. (D) Immunohistochemistry for bipolar cells (OTX2). (E) Graph showing cell counts of OTX2+ cell proportions across genotypes. Results display the mean + SEM for n = 3 for each genotype. Statistics are the result of Unpaired t-tests. * p < 0.05. Scale bars: 100 µm. Data files for graphs available in S5 Data. (TIF) [file pbio.3003332.s002.tif]

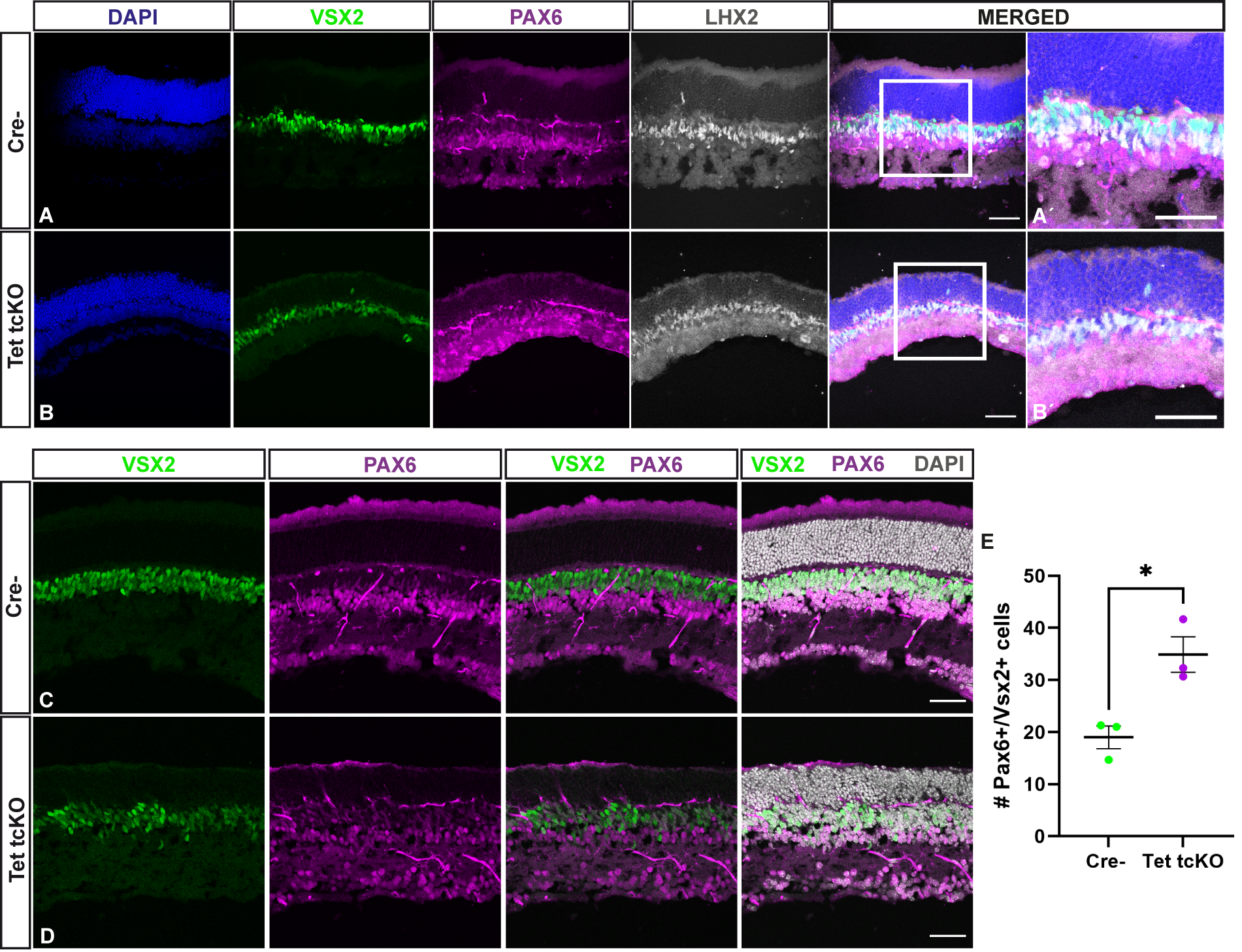

Supplement: S3 Fig — (A–D) Immunohistochemistry for bipolar cells (VSX2); amacrine cells (PAX6) and Müller glia cells (LHX2) markers in (A, C) Cre− and (B, D) Tet tcKO retinas indicating prominent co-localization of VSX2, PAX6, and LHX2 and in presumptive Müller glia in Tet tcKO retinas. (E) Graph showing the number of VSX2, PAX6 double positive cells across control and Tet tcKO retinas. Results display the mean + SEM for n = 3 for each genotype. Statistics are the result of an Unpaired t test. * p < 0.05. Scale bars: 100 µm. Data files for graphs available in S6 Data. (TIF) [file pbio.3003332.s003.tif]

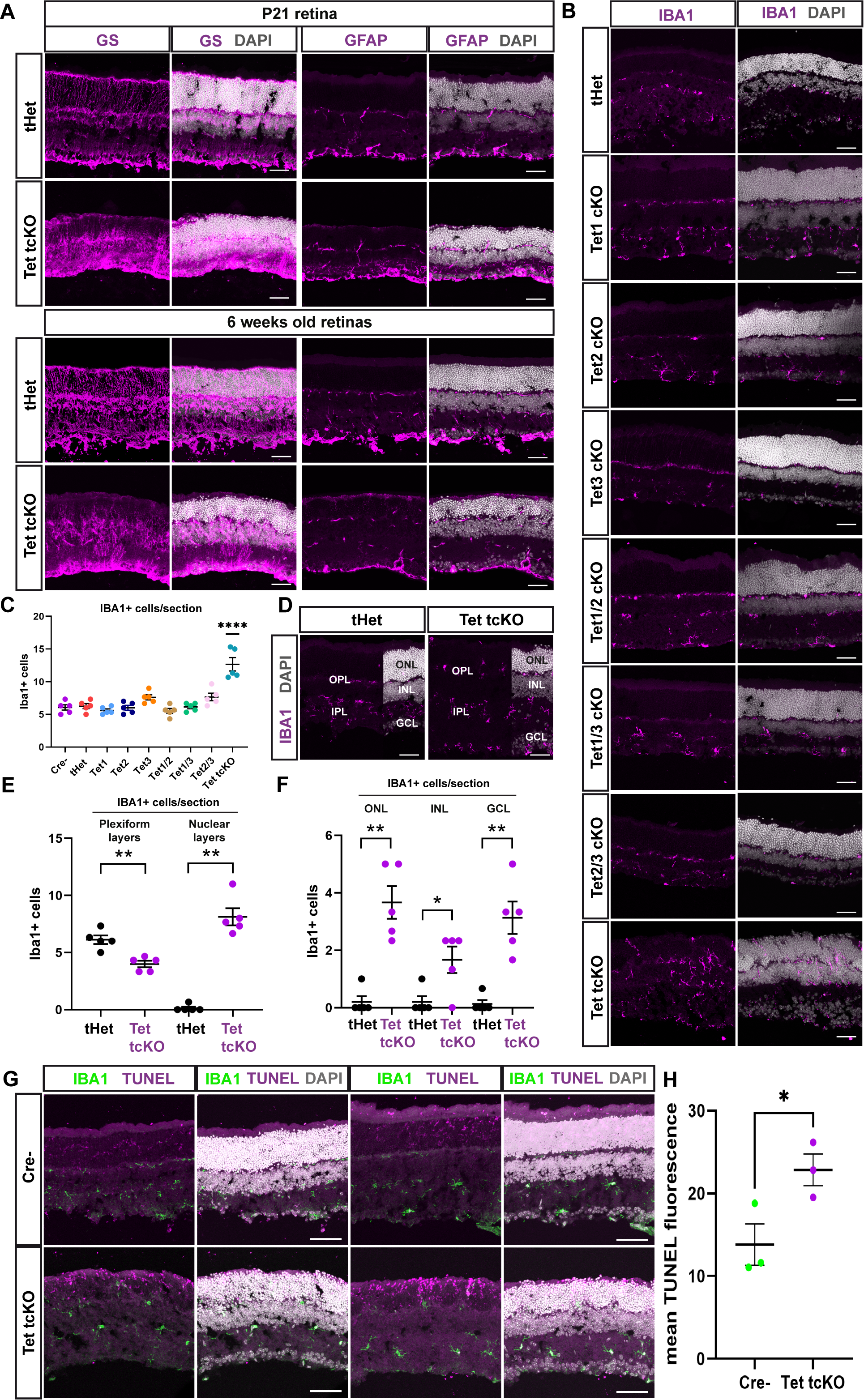

Supplement: S4 Fig — (A) Immunohistochemistry for Müller glia cells (GS and GFAP) in P21 and 6 weeks old retinas. (B) Immunohistochemistry for microglial cells (IBA1) show changes in cell proportions of microglial cells when TET enzymes are absent. (C) Results display the mean + SEM for n = 5 for each genotype. Statistics are the result of an Ordinary One-Way ANOVA, followed by a Dunnett’s multiple comparisons test. **** p < 0.0001. (D) Immunohistochemistry for microglial cells (IBA1) showing the comparisons between tHet and Tet1/2/3 cKO retinas and the layers that were considered for cell counts in E and F. (E, F) Graphs showing the difference in microglial cell localization in tHet and Tet tcKO retinas. Results display the mean + SEM for n = 5 for each genotype comparing (E) combined plexiform or nuclear layers, and (F) microglia localization within individual nuclear layers (ONL, INL, and GCL). Statistics are the result of an Unpaired t test or Mann–Whitney test. (E) ** p < 0.01. (F) * p < 0.05; ** p < 0.01. (G) Immunohistochemistry for microglia cells (IBA1) and apoptotic cells (TUNEL) in P21 Cre− and Tet tcKO retinas. (H) Results display the mean + SEM for n = 3 for each genotype. Statistics are the result of an Unpaired t test. * p < 0.05. Scale bars: 100 µm. Data files for graphs available in S7 Data. (TIF) [file pbio.3003332.s004.tif]

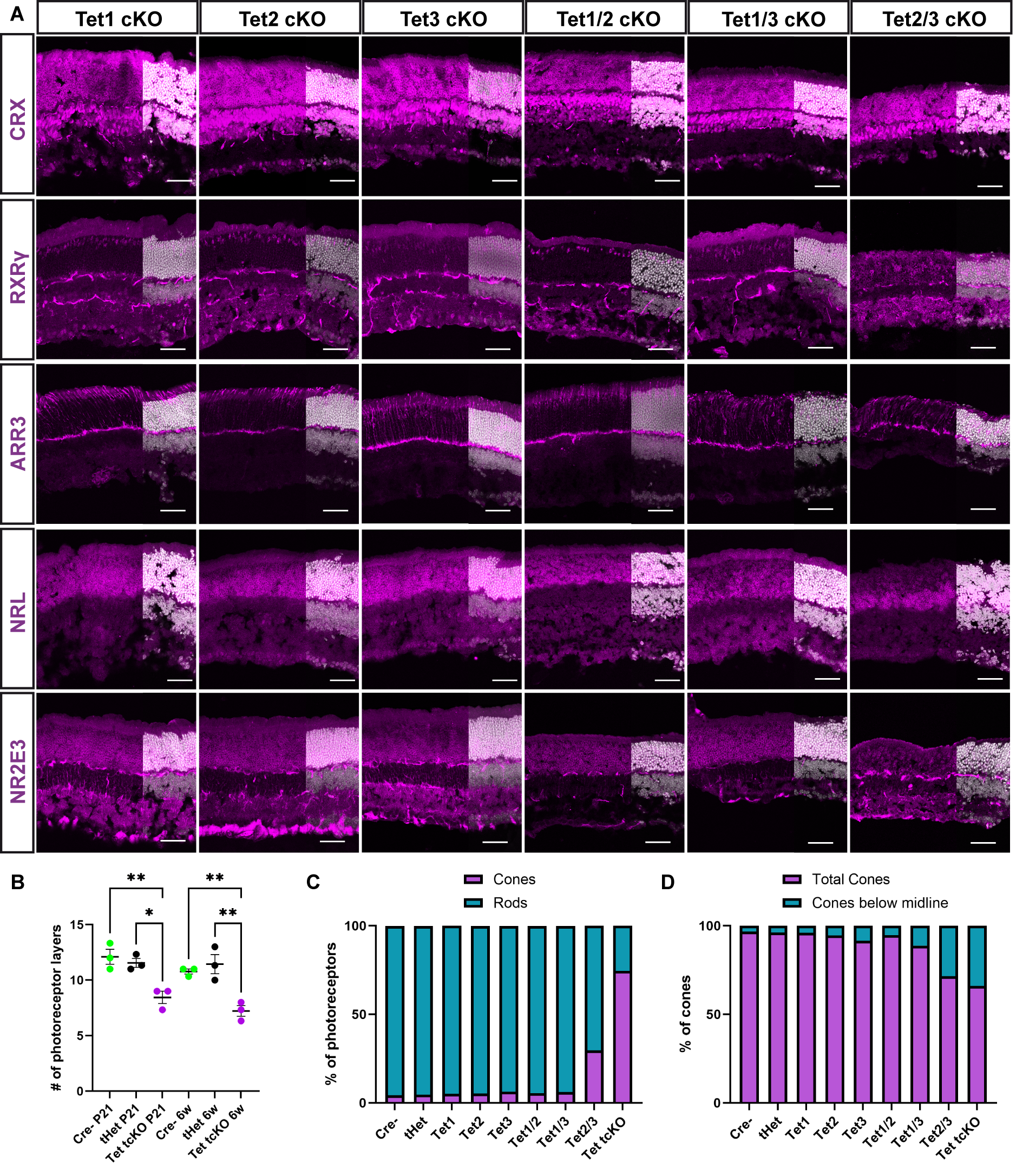

Supplement: S5 Fig — (A) Immunohistochemistry for cone-photoreceptor (CRX, RXRγ, and ARR3) and rod-photoreceptor (CRX, NRL, and NR2E3) markers indicate alterations in photoreceptor cells proportions when TET enzymes are absent. (B) Graphs showing the significant decrease in the number of photoreceptor cell nuclear layers in the ONL comparing Cre−, tHet, and Tet tcKO H&E-stained retinas both at P21 and 6 weeks. Results display the mean + SEM for n = 3 for each genotype. Statistics are the result of One-way ANOVA followed by a Tukey’s comparisons test * p < 0.05; ** p < 0.01. (C) Graphs showing the contribution of rod-photoreceptors and cone-photoreceptors to the total number of photoreceptor cells across genotypes. (D) Changes in the location of cone-photoreceptor nuclei in the ONL across genotypes, indicating presence of cone nuclei below the midline of the ONL. Scale bars: 100 µm. Data files for graphs available in S3 Data. (TIF) [file pbio.3003332.s005.tif]

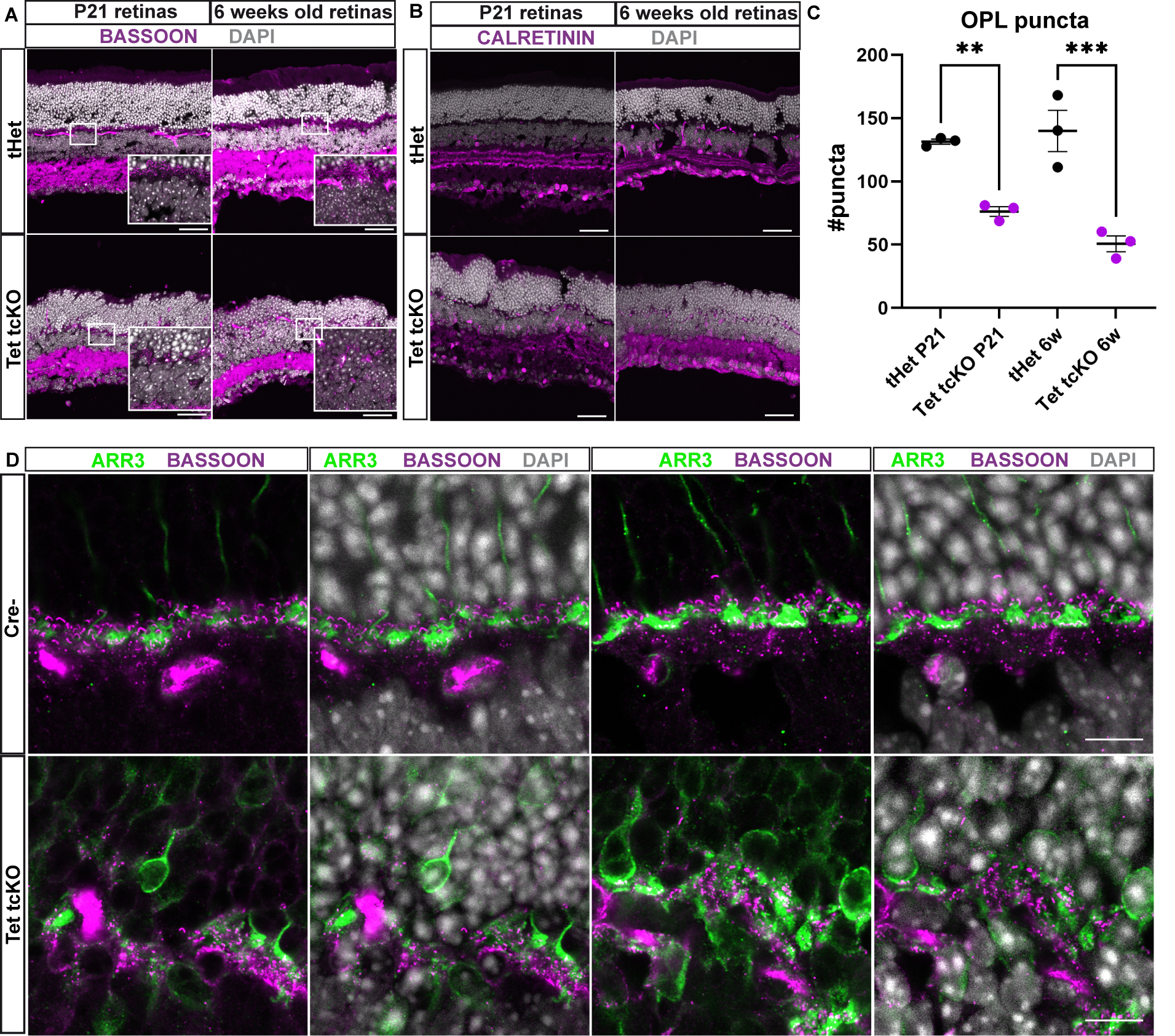

Supplement: S6 Fig — Tet tcKO results in altered retinal synapse structures. Related to Fig 1E. (A, B) Immunohistochemistry for synaptic markers BASSOON and CALRETININ that show the disruption of the OPL and IPL respectively, at P21 and 6 weeks. (C) Graph showing the number of ribbon synapses in the OPL labeled by BASSOON. Results display the mean + SEM for n = 3 for each genotype. Statistics are the result of a One-Way ANOVA, followed by a Tukey’s comparisons test; ** p < 0.01; *** p < 0.001. (D) Immunohistochemistry for cone-photoreceptors (ARR3) and the synaptic marker BASSOON in P21 retinas showing the loss of cone ribbon synapses in Tet tcKO retinas. Scale bars: (A, B) 100 µm; (D) 10 µm. Data files for graphs available in S9 Data. (TIF) [file pbio.3003332.s006.tif]

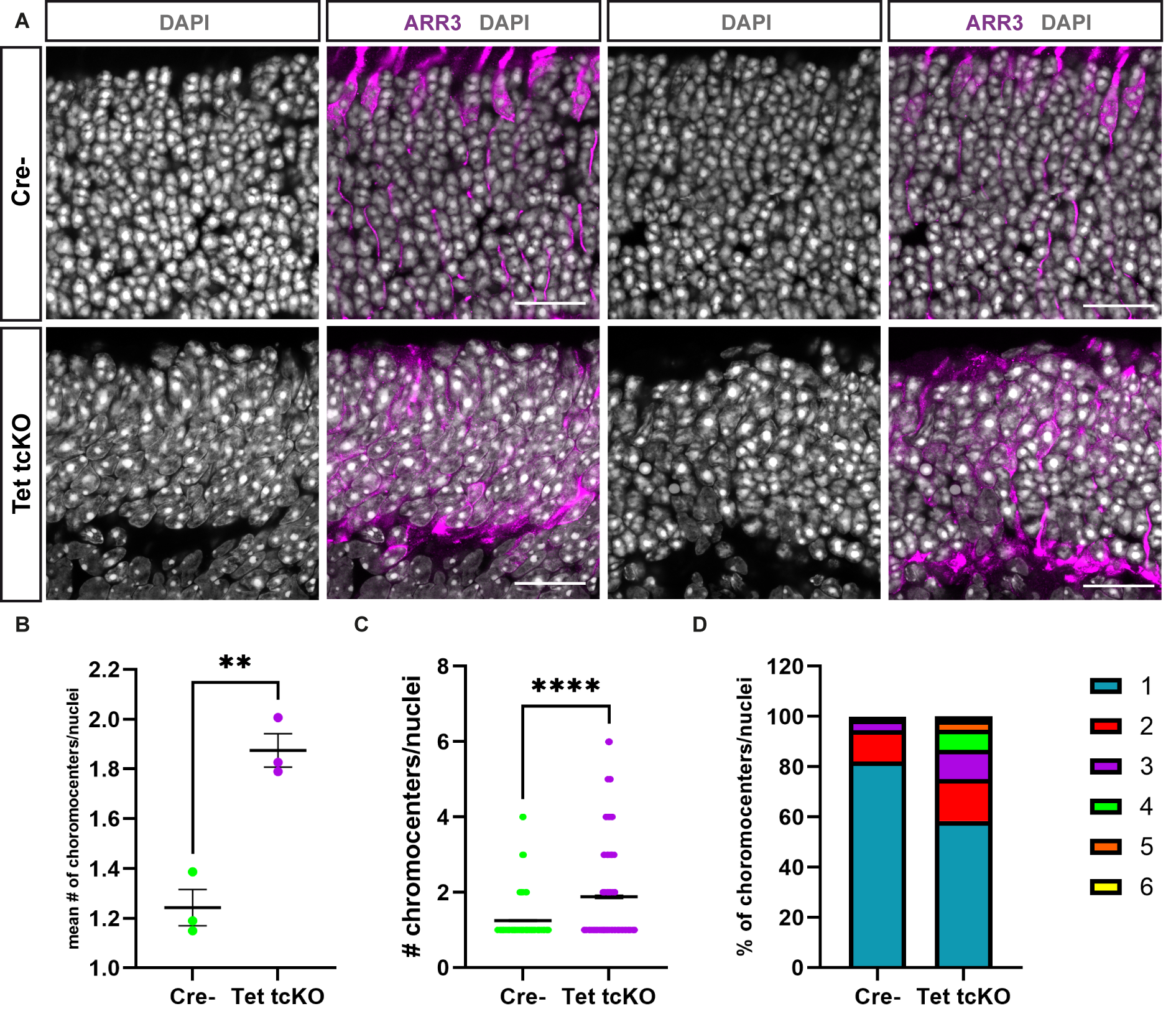

Supplement: S7 Fig — (A) Immunohistochemistry for nuclei (DAPI) and cone-photoreceptors (ARR3) in P21 retinas. (B) Graph showing the mean number of chromocenters/nuclei across genotypes. Results display the mean + SEM for n = 3 for each genotype. Statistics are the result of an Unpaired t test. ** p < 0.01. (C) Graphs showing the changes in the number of chromocenters/nuclei per genotype. Statistics are the result of a Mann–Whitney test. **** p < 0.0001. (D) Graphs showing the differences in proportion (%) of nuclei containing 1–6 chromocenters across genotypes. Scale bar: 20 µm. Data files for graphs available in S10 Data. (TIF) [file pbio.3003332.s007.tif]

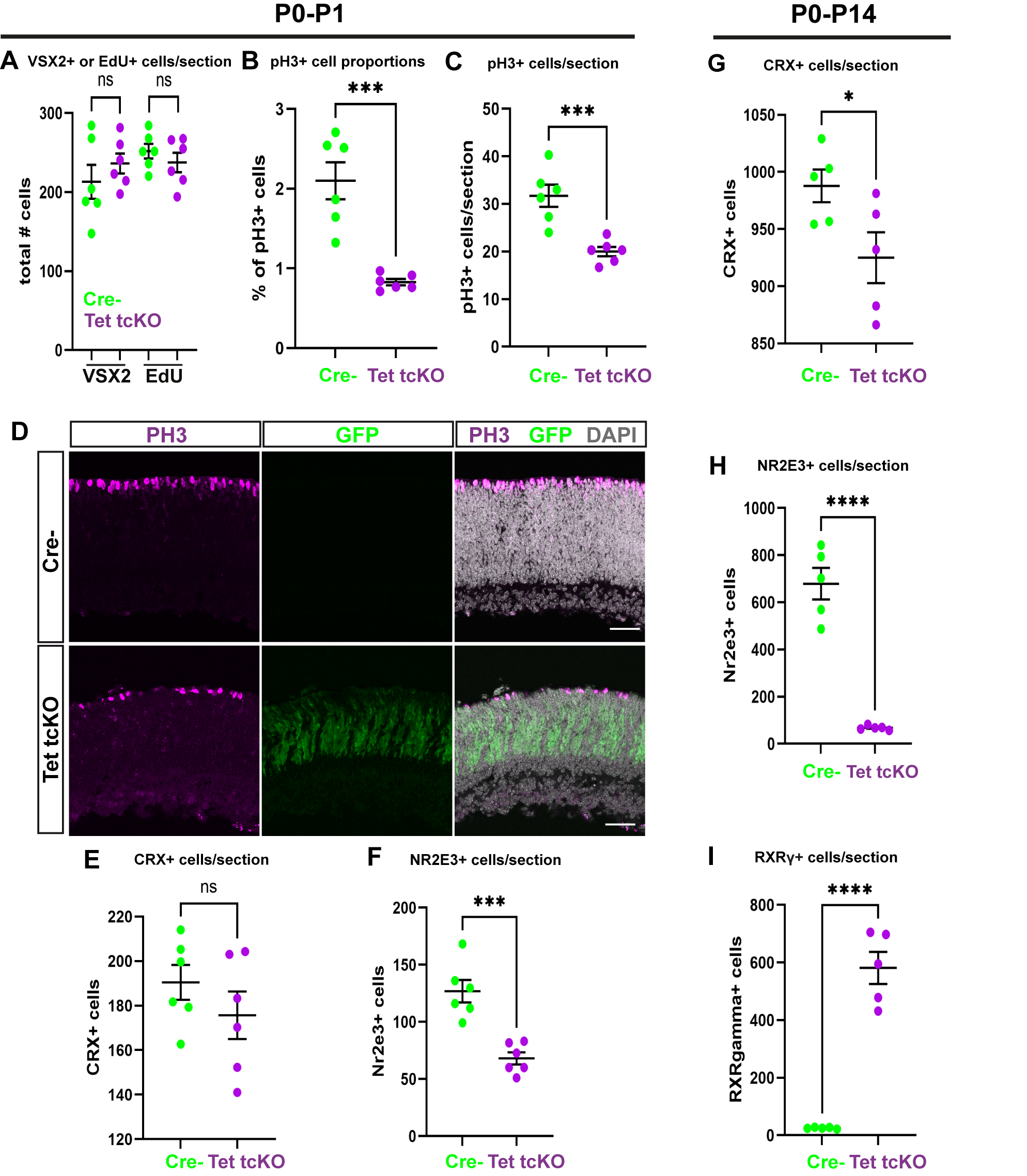

Supplement: S8 Fig — (A) Graph showing the total number of VSX2+ and EdU+ cells after P0-P1 EdU pulse. (B, C) Graphs showing the proportion (PH3+/EdU+) and total number of PH3+ cells P0–P1 EdU pulse. (D) Immunohistochemistry showing the labeling of mitotic cells (PH3+ cells). (E, G) Graphs showing the total number of CRX+ cells after (E) P0-P1 and (G) P0-P14 EdU pulses. (F, H) Graphs showing the total number of NR2E3+ and EdU+ cells after (F) P0–P1 and (H) P0–P14 EdU pulses. (I) Graph showing the total number of RXRγ+ cells after P0–P14 EdU pulse. Results display the mean + SEM for n = 5 (P0-P14) or n = 6 (P0-P1) for each genotype. Statistics are the result of two-tailed Unpaired t test; ns: nonsignificant; * p < 0.05, *** p < 0.001; **** p < 0.0001. Scale bars: 100 µm. Data files for graphs available in S12 Data. (TIF) [file pbio.3003332.s008.tif]

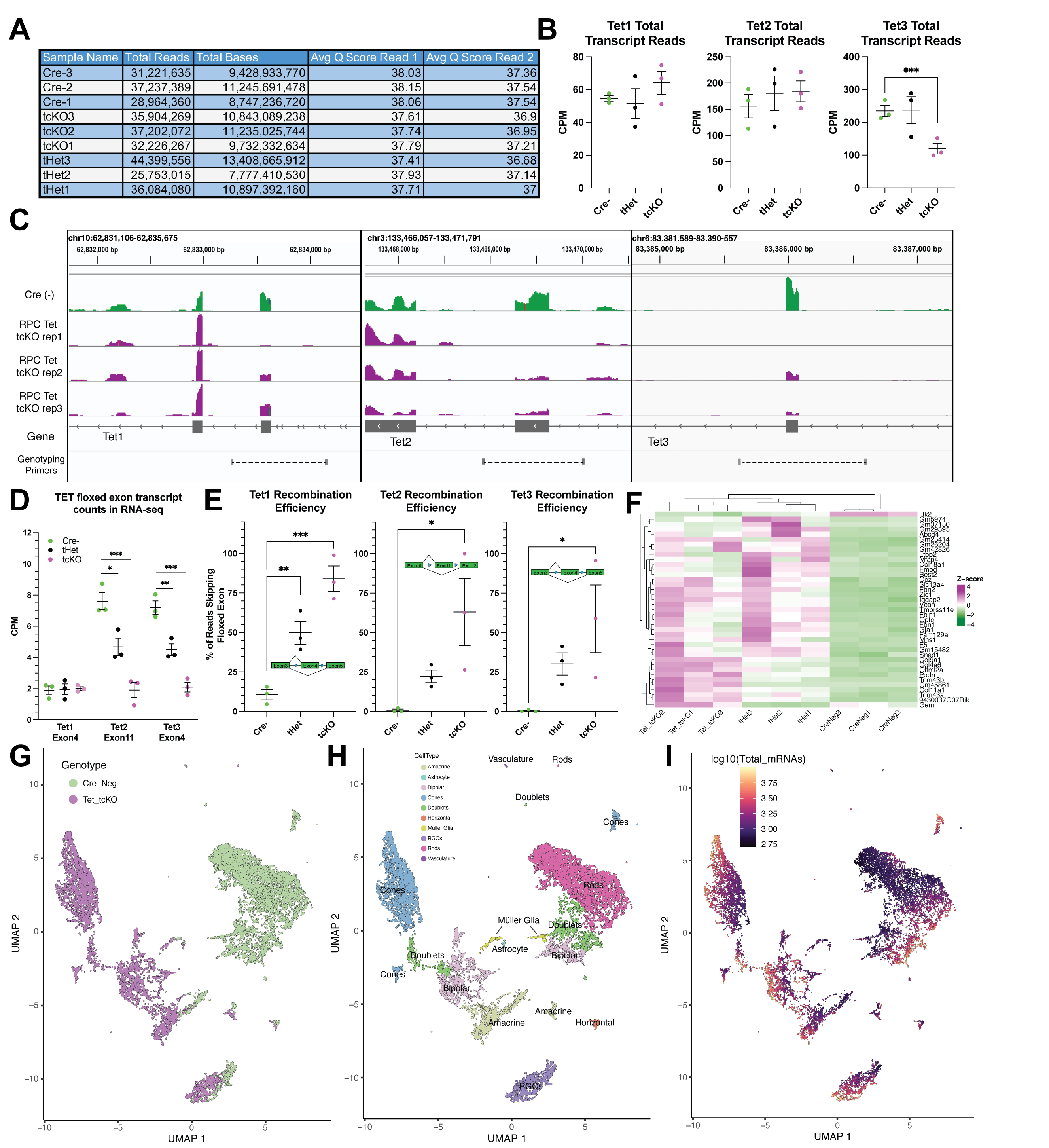

Supplement: S9 Fig — (A) Read depth and quality score metrics for bulk RNAseq samples. (B) Total transcript reads for Tet1 (left), Tet2 (middle) and Tet3 (right) across genotypes. (C) Genome browser tracks indicating read alignment to floxed exons in Cre− controls (green) and Tet tcKO replicates (purple) for Tet1 (left), Tet2 (middle), and Tet3 (right). (D) Total counts per million (CPM) for reads aligning to floxed exons across genotypes. (E) Splicing efficiency of floxed exons for Tet1 (left), Tet2 (middle), and Tet3 (right). Splicing efficiency is determined by the ratio of the number of reads that splice into the floxed exon divided by the sum of the splice reads including and excluding the floxed exon. (F) Heatmap of all differentially expressed transcripts (Fold Change > 2; q-value < 0.01) between Cre− and tHet RNAseq pairwise comparisons across all RNA-seq replicates. (G–I) UMAP dimension reductions of the full snRNAseq datasets with cells colored by (G) genotype, (H) annotated cell type, and (I) total transcripts detected per cell. Data files for graphs available in S13 Data. (TIF) [file pbio.3003332.s009.tif]

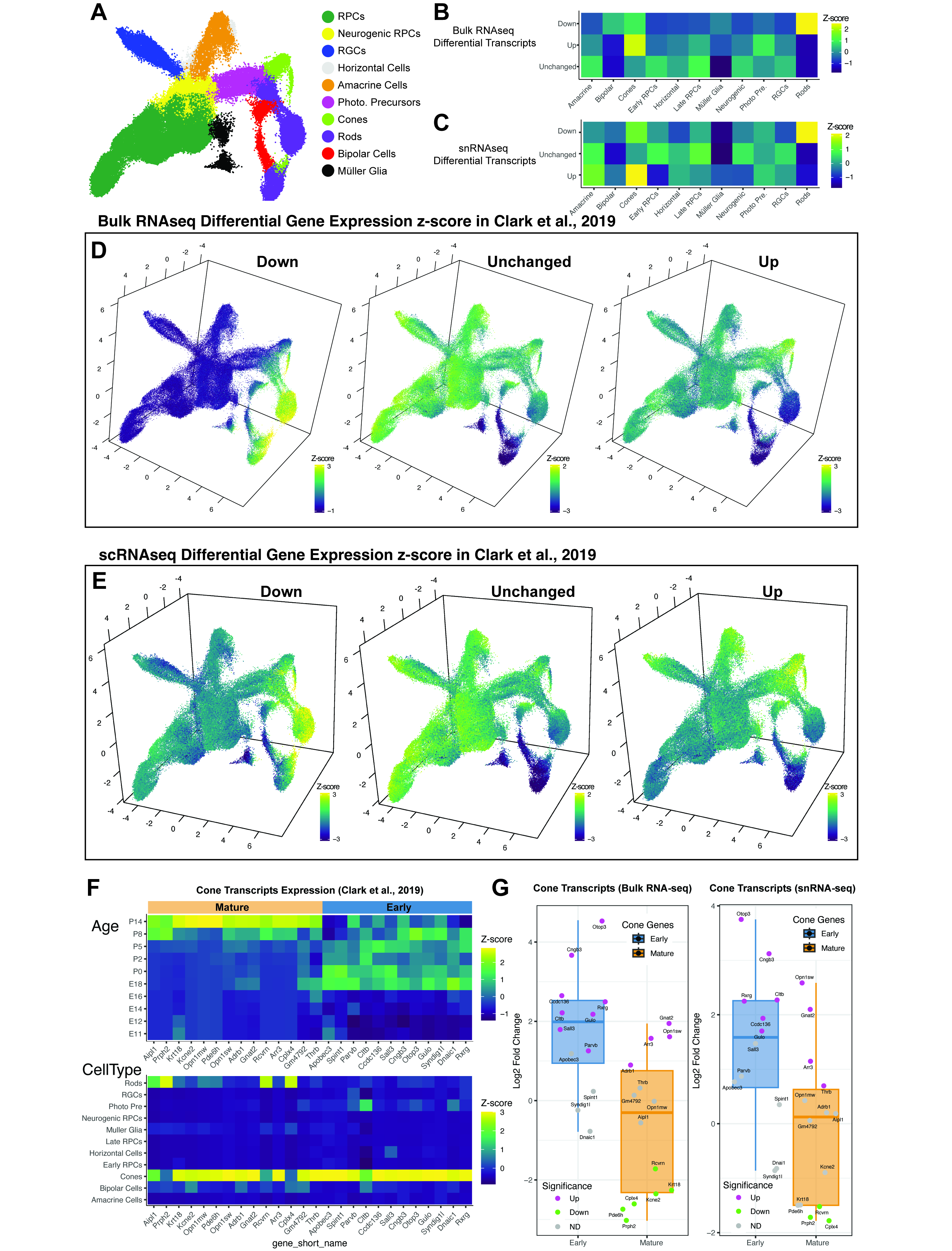

Supplement: S10 Fig — Related to Fig 5. Gene modules of differentially expressed genes highlight changes in photoreceptor gene expression patterns. (A) UMAP dimension reduction of the mouse retinal development scRNAseq dataset from [14] with cells colored by annotated cell type. (B, C) Heatmaps displaying relative z-scores of differentially expressed transcript gene modules from (B) P21 Tet tcKO RNAseq and (C) P21 Tet tcKO snRNAseq across annotated cell types in the mouse retinal development scRNAseq dataset. (D, E) UMAP dimension reductions of the [14] dataset with cells colored by relative z-scores of differentially expressed transcripts as gene modules from (D) P21 Tet tcKO RNAseq and (E) P21 Tet tcKO snRNAseq datasets. (F) Heatmaps of cone photoreceptors transcripts that display developmentally regulated expression enrichment across (top) developmental age and (bottom) retinal cell types from [14]. (G) Boxplots of the log2 fold-change of designated developmental (Early) or mature cone transcripts in Tet tcKO bulk RNA-seq and snRNA-seq datasets compared to Cre− controls, highlighting the increased expression of developmental cone transcript expression in Tet tcKO retinas. Data files for graphs available in S15 Data. (TIF) [file pbio.3003332.s010.tif]

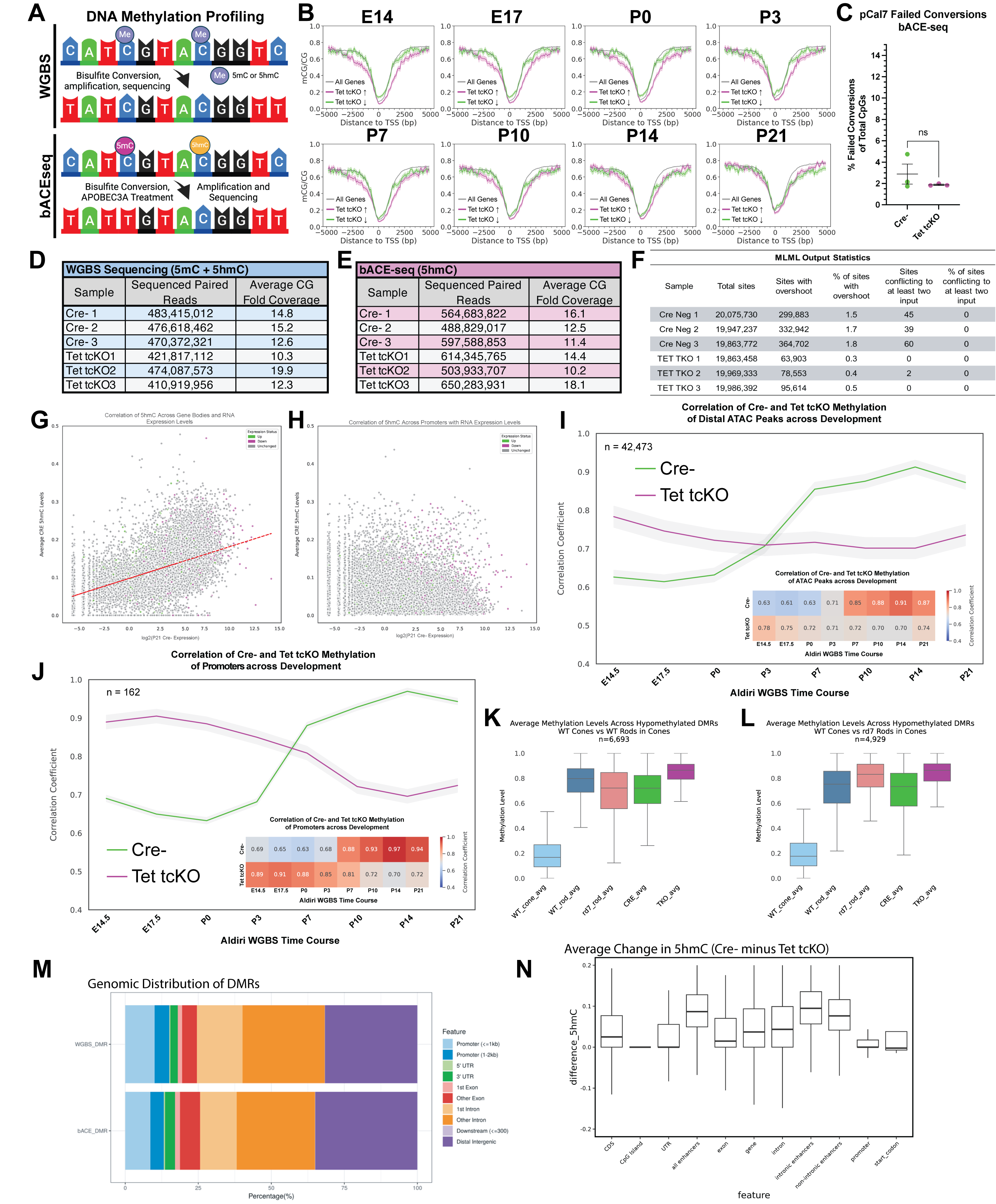

Supplement: S11 Fig — (A) Graphic displaying results of WGBS and bACE-seq to distinguish 5mC and 5hmC marks. (B) Comparisons of the temporal WGBS methylation patterns across retinal development within the ±5kb of the TSS for up- and down-regulated transcripts from Tet tcKO RNAseq experiments. (C) Graph highlighting estimated APOBEC3A failed conversion rates utilizing pCal7 plasmid spiked-in to each bACE-seq reaction. Statistics represent results of a student t test (ns, not significant). (D, E) Summary statistics for WGBS and bACE-seq experiments indicating total reads and average coverage for each sample. (F) MLML Output statistics for distinguishing 5hmC and 5mC marks. (G, H) Scatterplots assessing the correlation RNA expression levels and average 5hmC levels across the (G) gene body or (H) promoter regions for all genes. Individual genes are colored by differential expression in P21 RNA-seq comparisons between P21 Cre− and Tet tcKO retinas. (I, J) Line plots showing correlation coefficients of P21 Tet tcKO or Cre− control WGBS with temporal WGBS methylation profiles across (I) accessible DNA and (J) promoters for ATAC peaks that display a >10% decrease in methylation levels across development. (K, L) Boxplots of average methylation profiles for differentially hypomethylated regions identified in comparisons between (K) Cones vs. Rods and (L) Cones vs. rd7 Rods in P21 Cre− and TET tckO retinal samples and sorted cones, rods, and rd7 rods. (M) Genomic feature distribution for DMRs from WGBS and bACEseq analyses. (N) Boxplots of the change in 5hmC levels (Cre− minus Tet tcKO) between P21 Cre− and Tet tcKO retinal samples, highlighting the significant loss of enhancer methylation in Tet tcKO retinas. Data files for graphs available in S16 and S18 Data — https://doi.org/10.6084/m9.figshare.29575223.v1. (TIF) [file pbio.3003332.s011.tif]

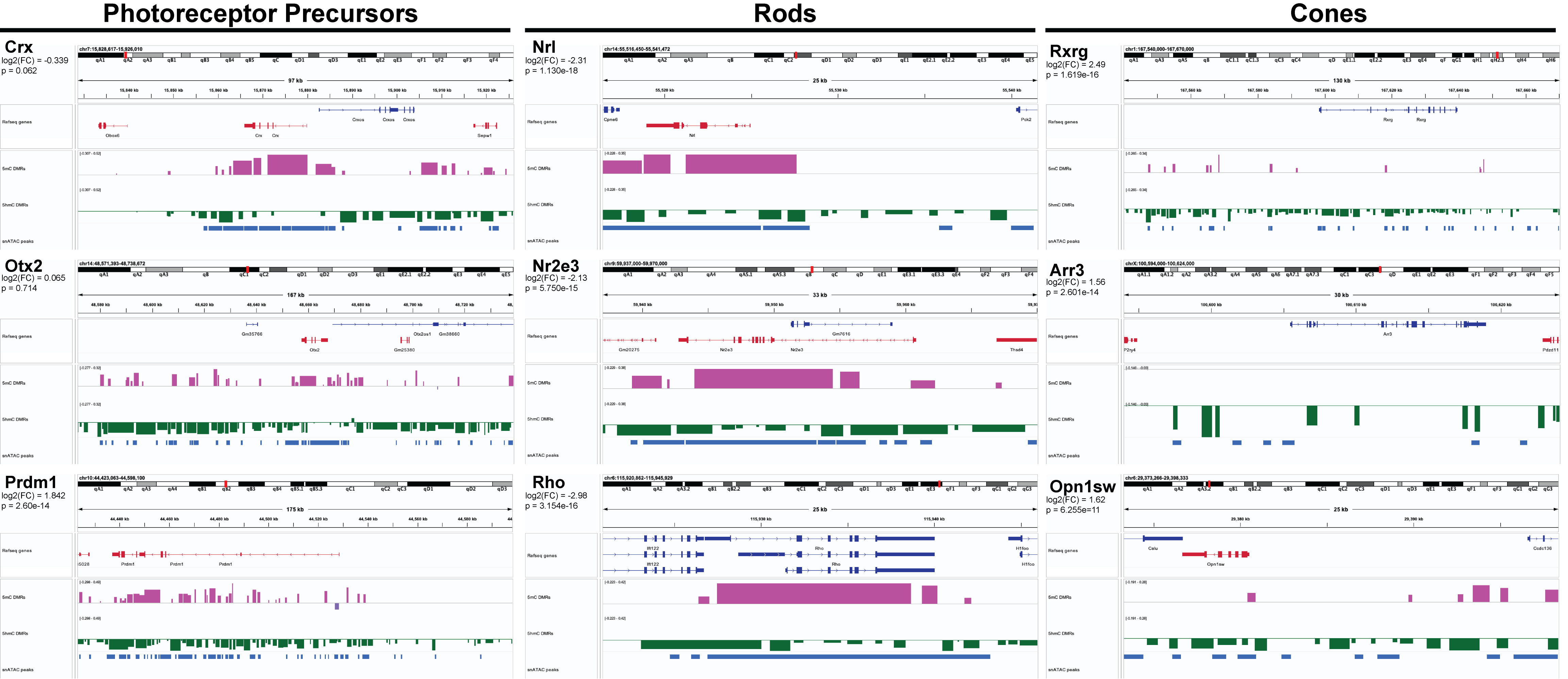

Supplement: S12 Fig — IGV genome tracks of photoreceptor transcription factor (left), rod photoreceptor (middle) and cone photoreceptor (right) gene loci. DMRs are shown for 5mC (maroon) and 5hmC (green) with the height of the bar representing the average difference in methylation between Tet tcKO and Cre− WGBS and bACE-seq, respectively. 5mC and 5hmC tracks indicate direction of DMR, with bars above or below the gray equivalence lines in each track indicating gain or loss of methylation, respectively. snATAC peak track (bottom, blue) indicates called peaks from the retinal development snATAC-seq studies in [102]. (TIF) [file pbio.3003332.s012.tif]
